# Sharing conspiracy theories and staying in power: How leaders' false theories influence leadership perception

**DOI:** 10.1111/bjso.70088

**Published:** 2026-04-28

**Authors:** Shen Cao, Jan‐Willem van Prooijen, Mark van Vugt

**Affiliations:** ^1^ Department of Experimental and Applied Psychology Vrije Universiteit Amsterdam Amsterdam the Netherlands

**Keywords:** adaptive conspiracism hypothesis, conspiracy theory, error management theory, evolutionary social psychology, leader perception

## Abstract

Research shows that spreading conspiracy theories impacts leaders' reputations; yet, it remains unclear how leaders are viewed when their theories are debunked. Across four studies (*N* = 1437), we explored whether conveying a conspiracy theory, regardless of its accuracy, influences followers' impressions of leader dominance, competence and warmth. Participants evaluated leaders who either incorrectly perceived (false‐positive) or incorrectly misperceived (false‐negative) a conspiracy about the cause of a simulated crisis. During intergroup conflict, false‐positive leaders were seen as less warm, similarly competent, yet more dominant than false‐negative leaders. The dominance gap grew when the consequences of overlooking a conspiracy were more severe. Conversely, in the absence of conflict, false‐positive leaders were perceived as less warm and competent than false‐negative leaders. These findings support an error management approach to conspiracy theories: Leaders who spread conspiracy theories, even if later debunked, are still perceived as strong leaders, particularly in conflict settings.

## INTRODUCTION

In recent years, public support for political leaders with dominant behaviours and personalities has grown, as seen with leaders like Donald Trump, Vladimir Putin, Narendra Modi and Jair Bolsonaro (Bakker et al., 2016; Jiménez et al., 2021; Petersen & Laustsen, 2020). These leaders often employ dominance strategies to retain power, including aggression, manipulation and deception, which generate fear, mistrust and anxiety (Redhead et al., 2019). A key manipulative tactic is inventing and spreading conspiracy theories, which are defined as beliefs that multiple actors secretly collude to achieve harmful goals (Bale, 2007). Conspiracy theories discredit opponents and consolidate support for the leader (Chigwedere et al., 2008; Plenta, 2020; Ren et al., 2022). For example, Donald Trump's claim that the 2020 US presidential election was stolen spurred some of his followers to storm the Capitol in January 2021.

Voters' political choices rely on their personal preferences for leader traits; thus, these perceptions serve as a fundamental mechanism explaining the shifting dynamics of political power (e.g., Kakkar & Sivanathan, 2017; Laustsen, 2021; Nai et al., 2021). Recognizing this, political candidates actively manage their public image and undermine opponents to strategically manipulate how voters perceive them (Laustsen, 2021). Therefore, to understand contemporary leader–follower dynamics, unpacking how followers' trait perceptions are formed and how leaders exploit them is critical. Spreading conspiracy theories influences perceptions of those who endorse them (Cao et al., 2025; Green, Toribio‐Flórez, Douglas, Brunkow, & Sutton, 2023). Whereas endorsing such theories can harm reputations by making leaders seem gullible (Cassam, 2016) or even untrustworthy (Green, Toribio‐Flórez, Douglas, Brunkow, & Sutton, 2023), it can also increase their perceived dominance, a desirable leadership trait especially in intergroup conflict (Laustsen & Petersen, 2017). This suggests that conspiracy theories can serve as a strategic tool for leaders to emphasize their dominance, albeit at the cost of being seen as less warm and competent (Cao et al., 2025). Thus, spreading conspiracy theories may benefit leaders by signalling assertiveness and aggression while risking reputation damage and potential loss of support if the theories are debunked (Maxey, 2021).

So far, the role of conspiracy theory accuracy in shaping leader perceptions largely remains unexplored in research. Because conspiracy theories can be proven true or false, it is important to understand how accuracy impacts leaders' reputations. This study examines how leaders' reputations change when their conspiracy theories are proven to be true or false. Based on insights drawn from evolutionary social psychology, we propose that spreading conspiracy theories decreases leaders' perceived warmth but increases their perceived dominance, even when proven false.

### The adaptive conspiracism hypothesis and error management theory

From an evolutionary perspective, the human tendency to endorse and spread conspiracy theories may reflect an underlying capacity to detect genuine conspiracies. The adaptive conspiracism hypothesis (ACH; van Prooijen & van Vugt, 2018) proposes that ancestral environments were characterized by recurrent threats from hostile coalitions. In such contexts, the ability to anticipate and detect conspiratorial intent—alliances formed to harm, exploit or outcompete one's group—would have conferred substantial survival and reproductive advantages. Anthropological and primatological evidence suggests that coalitional aggression was a persistent feature of human evolutionary history and a major source of mortality (Knauft et al., 1987; Wrangham, 1999).

Under these conditions, natural selection would have favoured a cognitive system biased towards over‐detection rather than under‐detection of conspiracies. Missing a real conspiracy (a false negative) could be fatal, whereas falsely inferring one (a false positive) is typically less costly. As a result, humans may have evolved a systematic tendency to overestimate the likelihood that others are coordinating against them.

This logic is formalized in error management theory (EMT; Haselton & Buss, 2000; Johnson et al., 2013), which explains how natural selection shapes decision‐making under asymmetric costs. When the consequences of different types of errors are unequal, cognitive systems evolve to minimize the more costly error, even at the expense of increasing less costly ones. A familiar biological analogy is the immune system: allergic responses represent false alarms, yet they persist because failing to detect genuine pathogens would be far more dangerous. The same principle applies across psychological domains. In mating contexts, for instance, women tend to underestimate men's commitment, whereas men tend to overestimate women's sexual interest, biases that reflect sex‐specific asymmetries in reproductive costs (Haselton & Buss, 2000). More broadly, EMT has been used to explain systematic biases in perception, judgement and intergroup behaviour (McKay & Efferson, 2010).

Applied to conspiracism, EMT suggests that humans possess a bias towards false positives in conspiracy detection, erring on the side of suspicion to avoid potentially catastrophic oversight. However, detecting a conspiracy is only half the adaptive problem. Unlike physiological defences or individual mating decisions, the benefits of conspiracy detection depend on collective action. A perceived threat becomes evolutionarily relevant only if it can be communicated and acted upon by others.

This introduces a crucial social layer: individuals must persuade and mobilize their coalition. The act of spreading conspiracy narratives can therefore be understood not merely as belief expression, but as an attempt at coalitional coordination, that is, aligning attention, shaping threat perception and motivating a group response. In this sense, conspiracism intersects with the evolved functions of leadership, which centre on solving collective action problems under conditions of threat and uncertainty (van Vugt & Grabo, 2015).

### Leader–follower dynamics in conspiracy detection

From an evolutionary perspective, leadership emerges to solve collective action problems by providing public goods, the benefits that enhance group survival but are costly for individuals to produce (Price & van Vugt, 2014). One such public good in contexts of intergroup conflict is the early detection of conspiracies. By identifying potential threats before they fully materialize, leaders help coordinate defensive action and reduce the risk of collective harm.

Yet this function creates a fundamental dilemma. The cues that trigger suspicion of a conspiracy are often ambiguous, incomplete and asymmetrically distributed: leaders may detect weak signals that are not directly observable to most followers. As a result, warnings about hidden threats can appear unsubstantiated or exaggerated to the group. What is, from the leader's perspective, a prudent false alarm may, from the follower's perspective, resemble manipulation or even paranoia.

This asymmetry generates substantial reputational risks for leaders. Publicly accusing an outgroup of conspiratorial intent, especially when evidence is uncertain, can violate social norms of fairness and restraint. Empirically, individuals who endorse conspiracy theories are often stigmatized, perceived as less credible and sometimes socially excluded (Green, Toribio‐Flórez, & Douglas, 2023; Green, Toribio‐Flórez, Douglas, Brunkow, & Sutton, 2023; Harambam & Aupers, 2017; Lantian et al., 2018). Repeated false alarms further erode trust through a classic ‘cry wolf’ dynamic (Breznitz, 2013), undermining cooperation within the group and credibility in intergroup relations (Cassam, 2016; Klein et al., 2015). In short, while false negatives are dangerous for the group, false positives are costly for the leader.

This tension is resolved, in part, through the logic of service‐for‐prestige reciprocity (Price & van Vugt, 2014). Leaders who incur personal costs to provide valuable public goods are rewarded by followers with status, respect and continued support. Applied to conspiracy detection, this implies that followers face a trade‐off: If they punish leaders for every false positive, leaders will rationally lower their sensitivity to potential threats, thereby increasing the risk of missing genuinely dangerous conspiracies.

To prevent this, followers are incentivized to tolerate a degree of over‐detection. Occasional false alarms can be reframed not as incompetence, but as evidence of vigilance and commitment to group protection. In this sense, a leader's heightened sensitivity to conspiracies may function as a costly signal of ingroup loyalty (Grabo et al., 2017). By granting prestige despite occasional errors, followers effectively ensure themselves against catastrophic oversight. Consequently, the persistence of conspiracy signalling within groups reflects not only individual cognitive biases, but also a co‐evolved leader–follower dynamic: leaders err on the side of suspicion to protect the group, while followers absorb some of the social costs to maintain this protective function.

### Signalling resistance: Dominance as reputational compensation

Service‐for‐prestige theory assumes that leaders who provide public goods are typically rewarded with prestige, a form of status grounded in respect, admiration and voluntary deference (Price & van Vugt, 2014). However, we argue that the specific service of conspiracy detection follows a different reputational logic. Rather than primarily eliciting prestige, it is often compensated through the attribution of dominance.

Dominance, as conceptualized within the interpersonal tradition (Wiggins, 1979), reflects a strategy centred on power, assertiveness and a willingness to coerce. When leaders publicly accuse an outgroup of conspiratorial intent, they do more than signal vigilance. They also display a readiness to confront hidden enemies, take risks and act decisively under uncertainty. These behaviours, particularly when expressed in the absence of definitive evidence, carry reputational costs but simultaneously serve as signals of formidability.

This dual nature is critical. On the one hand, conspiracy accusations expose leaders to potential backlash if proven unfounded. On the other hand, they communicate traits that are evolutionarily valued in conflict contexts: toughness, resolve and a low threshold for threat response. Consistent with this interpretation, empirical work shows that individuals who endorse or spread conspiracy theories are perceived as more dominant than those expressing neutral views (Cao et al., 2025), particularly under conditions of intergroup tension.

From this perspective, dominance attributions can be understood as a form of reputational compensation for the risks leaders incur. Drawing on the Adaptive Followership framework (Laustsen, 2021; Laustsen & Petersen, 2017), follower preferences are highly context‐sensitive. In peaceful or stable environments, prestige‐based traits (i.e., competence, fairness and prosociality) tend to be prioritized. In contrast, under perceived threat, followers shift towards leaders who signal an ability to confront and neutralize adversaries. Dominant leaders are particularly well‐suited to these conditions. They are perceived as more capable of enforcing coordination, deterring rivals and reducing uncertainty in volatile environments (Halevy et al., 2012; Spisak et al., 2015).

Consequently, when leaders exhibit heightened sensitivity to potential conspiracies, even at the risk of false positives, followers may respond not by withdrawing support but by endorsing and amplifying their dominance. In this way, conspiracy signalling becomes embedded in a broader leader–follower exchange: leaders incur reputational risk by sounding the alarm, and followers compensate by granting them a dominance‐based status that legitimizes decisive, and sometimes coercive, action.

### The present research

Building on the ACH and EMT, we proposed that the attribution of dominance functions as a form of reputational reciprocity towards leaders who engage in conspiracy detection, particularly when they commit false‐positive errors. Specifically, we hypothesized that in contexts of intergroup conflict, leaders who spread conspiracy narratives would be perceived as more dominant than leaders who communicate neutral explanations, even when those conspiracy claims are subsequently proven false.

**FIGURE 1:**
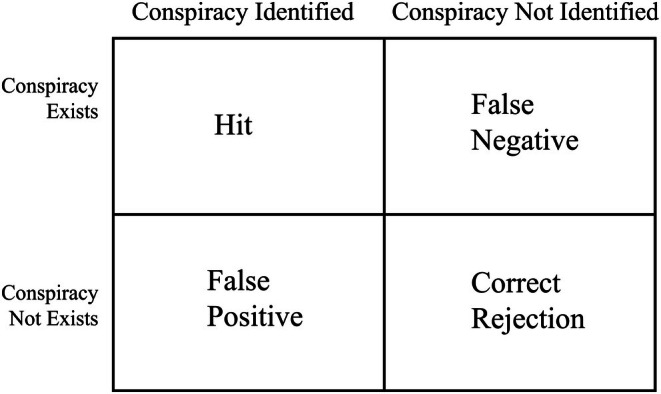
Error management theory in the context of conspiracy detection.

To test this hypothesis, we employed a series of scenario‐based experiments grounded in EMT. This framework is particularly relevant in environments where individuals must distinguish signal from noise, such as when evaluating the credibility of conspiracy claims. Within this paradigm (see Figure 1), four possible outcomes can be distinguished: a hit (correct detection of a real conspiracy), a correct rejection (accurately dismissing a non‐existent conspiracy), a false positive (erroneously inferring a conspiracy) and a false negative (failing to detect a real conspiracy). Crucially, EMT predicts that when the costs of these errors are asymmetric, as is typically the case in intergroup conflict, decision systems will be biased towards minimizing the more costly error. In this context, false negatives (failing to detect a genuine threat) are more dangerous for the group than false positives (wrongly suspecting a threat), which may nevertheless impose social and reputational costs.

Beyond dominance, we also assessed leader perceptions along the two fundamental dimensions of social cognition: competence (e.g., intelligence, skill, effectiveness; Fiske et al., 2007) and warmth (e.g., friendliness, trustworthiness; Cuddy et al., 2008). Prior research indicates that, in intergroup conflict, individuals who spread conspiracy theories are perceived as equally competent as those expressing neutral views, but as significantly lower in warmth (Cao et al., 2025).

Rather than interpreting this reduction in perceived warmth as a purely negative outcome, we conceptualize it as part of a strategic trade‐off between warmth and dominance in leader evaluation. Drawing on the fundamental follower needs framework (Sheng et al., 2026), different ecological conditions activate distinct follower priorities. Contexts requiring coordination and alliance‐building primarily activate the need for affiliation, favouring warm leaders. In contrast, contexts characterized by threat activate the need for protection, favouring dominant leaders. Notably, the need for expertise, and thus competence, appears to remain relatively stable across contexts.

From this perspective, the decreased warmth associated with conspiracy signalling does not necessarily undermine leadership. Instead, it may reflect an adaptive recalibration of follower preferences: when groups perceive external threats, they become more willing to trade warmth for dominance. By signalling vigilance towards hidden dangers, leaders may gain implicit authorization to act decisively, even aggressively, on behalf of the group.

Taken together, this reasoning leads to our central prediction: leaders who spread conspiracy theories will be perceived as more dominant than those who provide neutral accounts, particularly in intergroup conflict settings, and this effect will persist even when the conspiracy claims are false.

We tested this prediction across four studies in which participants imagined themselves as members of a group facing a crisis under the guidance of a leader. Study 1 examined leader evaluations across the full EMT matrix (false positives, false negatives, hits and correct rejections). Studies 2 to 4 extended this design by manipulating contextual features, including the presence versus absence of intergroup conflict and cooperative versus competitive environments, using both ancestral and modern scenarios. Studies 3 and 4 were preregistered on the Open Science Framework (OSF). Across all studies, we reported all manipulations, measures and exclusions. The research program was approved as part of a cluster application by the Scientific and Ethical Review Board of the Faculty of Behavioural and Movement Sciences at Vrije Universiteit Amsterdam.

## STUDY 1

Using a 2 (correct vs. false) by 2 (conspiracy vs. neutral) between‐subject scenario study, the first study aimed to explore how people perceive leaders under these four conditions when their group conflicts with another group. We hypothesized that: (1) leaders spreading conspiracy theories are perceived as more dominant and less warm compared to neutral leaders (H1.1); (2) leaders making correct (vs. false) inferences are perceived as more dominant, competent and warmer (H1.2); and (3) leaders spreading conspiracy theories are perceived as more dominant, competent and warmer when their theories are correct rather than false (H1.3).

### Method

#### Participants

All power analyses were conducted via G*power (version 3.1; Erdfelder et al., 2009). A minimum of 351 participants were required to achieve 80% power (with a small‐to‐medium‐sized effect for the interaction, effect size *f* = .15, α = .05). We recruited 400 participants (100 for each condition) from the United States via the Prolific platform. Twenty‐four of them were excluded because they failed the attention checks and the manipulation check, resulting in 376 eligible participants (183 males, 188 females and 5 others, ranging from 19 to 92 years old, *M* = 40.9, SD = 14.1).

#### Materials

**FIGURE 2:**
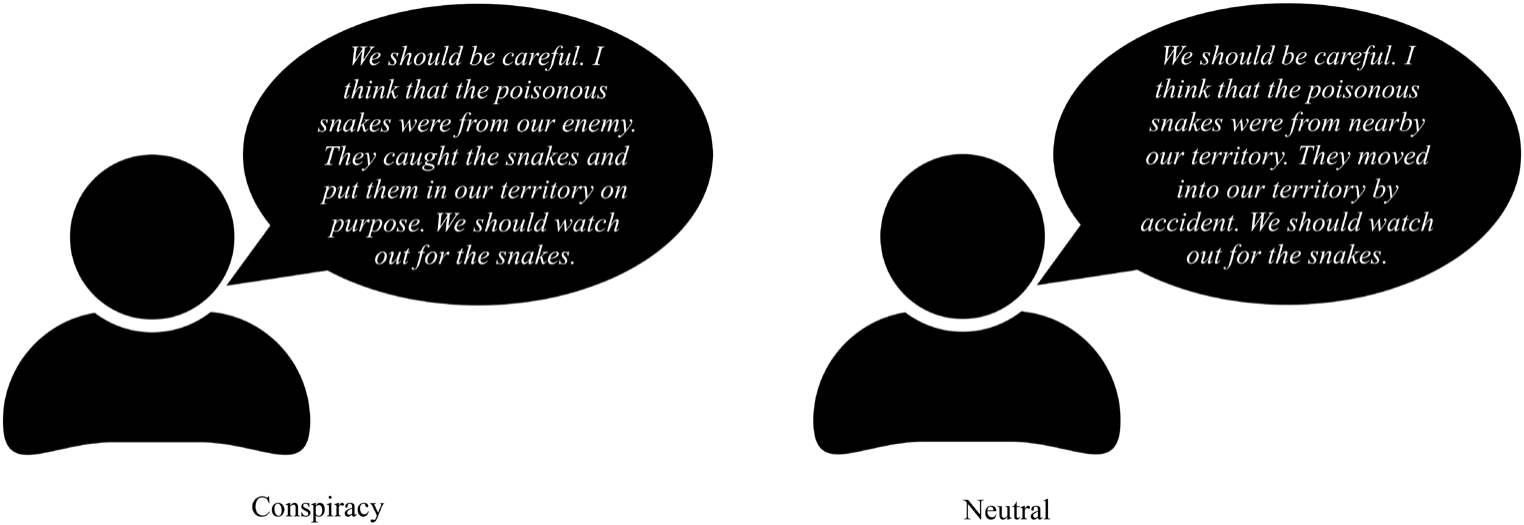
Conspiracy theory versus Neutral reason.

The comprehensive scenarios are available in the Online Supporting Information (OSM, https://osf.io/r2wx4/overview?view_only=4bb92b7a7a884fa98e7253bf66f1883a). Participants imagined living in a tribe in the Amazon Rainforest and engaging in competition with another tribe, thus keeping intergroup conflict constant (Cao et al., 2025). Recently, several members of their tribe succumbed to venomous snake bites. In line with signal detection theory, we established four conditions: (1) In the ‘Hit’ condition, the tribal leader, named Aru, claimed that the snakes were sent by the rival tribe (a conspiracy theory, as depicted in Figure 2). Participants read at the end of the materials that the leader's claim was proven to be true. (2) In the ‘False Positive’ condition, Aru proposed a conspiracy theory about the snakes, but participants read that the leader's claim was proven to be wrong, and the true reason was that snakes wandered into their territory by accident. (3) In the ‘Correct Rejection’ condition, Aru stated that the snakes had accidentally entered their territory, and participants read that the statement was confirmed. (4) In the ‘False Negative’ condition, Aru suggested that the snakes had arrived by accident, but participants read that it was a conspiracy orchestrated by the rival tribe. In each scenario, participants were asked to evaluate their perception of Aru.

#### Measurements

##### Perceived warmth

Perceived warmth was measured by five items from the Agreeableness subscale of the Big Five Inventory (John & Srivastava, 1999; e.g., ‘I think that Aru is helpful and unselfish with others’). The internal reliability was good (α = .91).

##### Perceived competence

Perceived competence was measured by the adapted version of The Self‐Competence Scale (five items rated on a 7‐point Likert scale, Tafarodi & Swann, 1995). The internal reliability was good (α = .89).

##### Perceived dominance

Perceived dominance was measured by the dominance subscale from the Mini‐markers of Evil questionnaire (Harms et al., 2007; Kim et al., 2020). There are five items (e.g., ‘I think Aru is dominant/forceful’) measured by a 7‐point Likert scale (1—‘strongly disagree’ to 7—‘strongly agree’). The internal reliability was good (α = .91).

#### Procedure

Participants were randomly assigned to one of the four conditions. After reading the scenario, participants responded to the trait‐impression questions, which were presented in a fixed order. There are two attention‐check questions asking participants to select a specific answer, as well as a manipulation check asking participants to choose what Aru proclaimed.

### Results

#### Descriptive results

The means, standard deviations and correlations are shown in Table 1.

**TABLE 1:** The means, standard deviations and correlations of measured variables (*N* = 376).

	*M*	*SD*	1	2	3
1. Age	40.9	14.1	‐		
2. Dominance	5.2	1.1	.08	‐	
3. Competence	4.9	1.2	−.11*	.58***	‐
4. Warmth	4.4	1.0	−.02	.13*	.45***

*Note*: *p < .05; ***p < .001.

#### Hypothesis testing

For perceived dominance, people rated leaders spreading conspiracy theories as more dominant (*M* = 5.5, *SD* = 0.9) than neutral ones (*M* = 4.9, *SD* = 1.1), *F* (1, 372) = 31.59, *p* < .001, η_p_^2^ = .08, *CI*_95%_ [0.03, 0.13]. Moreover, leaders making correct inferences were perceived as more dominant (*M* = 5.5, *SD* = 0.8) than those making false inferences (*M* = 4.9, *SD* = 1.2), *F* (1, 372) = 41.31, *p* < .001, η_p_^2^ = .10, *CI*_95%_ [0.05, 0.16]. A significant interaction also emerged, *F* (1, 372) = 12.72, *p* < .001, η_p_^2^ = .03, *CI*_95%_ [0.01, 0.08]. Multiple comparisons with Bonferroni adjustment suggested that perceived dominance between conspiracy leaders in the false positive (*M* = 5.3, *SD* = 1.0) condition did not differ significantly from the correct condition (hit; *M* = 5.6, *SD* = 0.8), *t*(372) = − 2.02, *p* = .267, which was not in line with H1.3. However, false‐positive leaders were considered more dominant than false‐negative leaders (*M* = 4.4, *SD* = 1.2), *t*(372) = − 6.47, *p* < .001. In contrast, when leaders were correct, the conspiracy‐spreading and neutral leaders (correct rejection; *M* = 5.4, *SD* = 0.8) were not significantly different, *t*(372) = − 1.56, *p* = .725. These results suggested that sharing conspiracy theories was the best strategy to gain dominance, which was in line with our theoretical line of reasoning.

**FIGURE 3:**
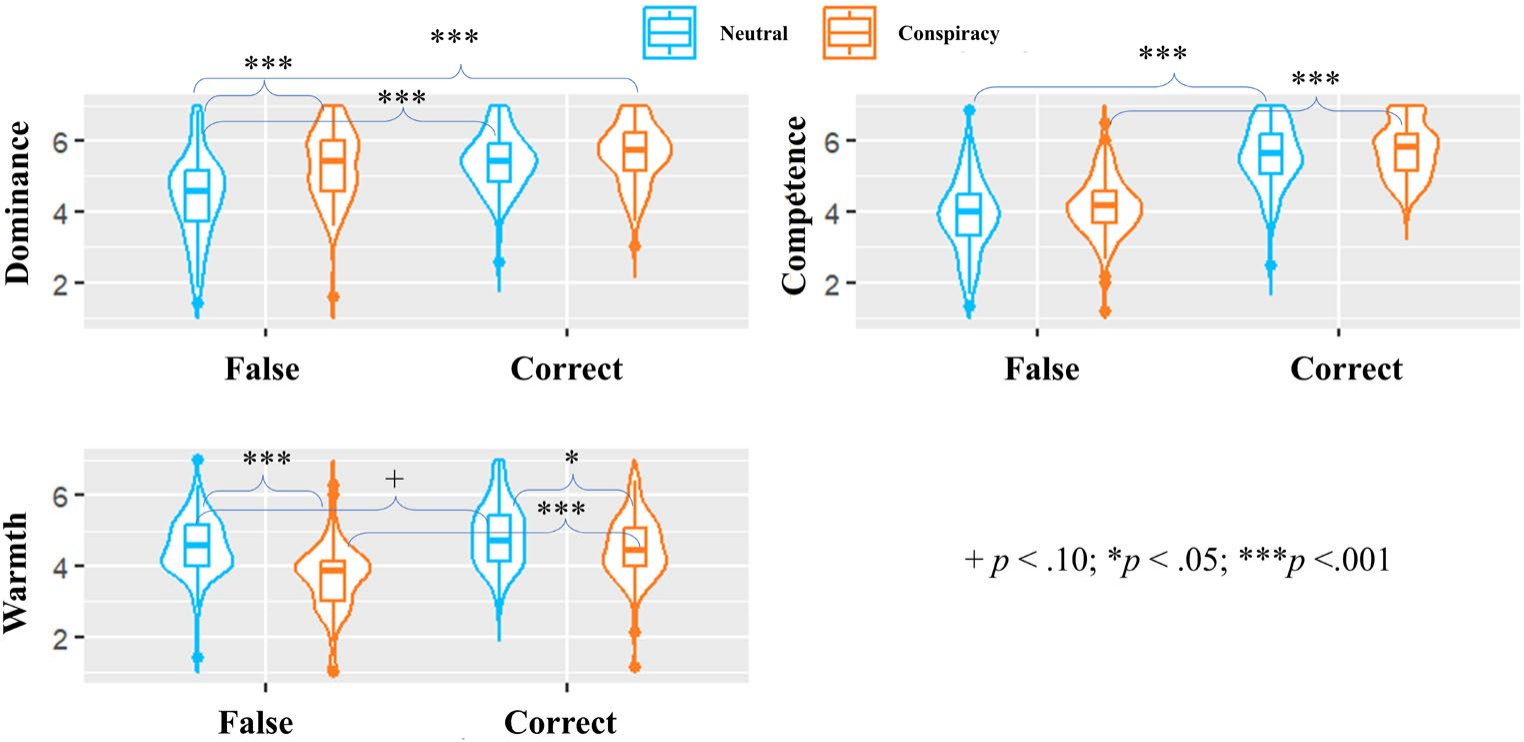
The results of the two‐way ANOVAs (Bonferroni corrected).

Leaders making false inferences were perceived as less warm (*M* = 4.1, *SD* = 1.0, *F* (1, 372) = 39.41, *p* < .001, η_p_^2^ = .10, *CI*_95%_ [0.05, 0.16]) and less competent (*M* = 4.0, *SD* = 1.0, *F* (1, 372) = 320.28, *p* < .001, η_p_^2^ = .46, *CI*_95%_ [0.39, 0.52]) than those making correct inferences (*M*_warmth_ = 4.7, *SD*_warmth_ = 0.9; *M*_competence_ = 5.7, *SD*_competence_ = 0.8). Moreover, people considered conspiracy leaders as less warm (*M* = 4.1, *SD* = 1.0) than the neutral ones (*M* = 4.7, *SD* = 0.9), *F* (1, 372) = 47.01, *p* < .001, η_p_^2^ = .11, *CI*_95%_ [0.06, 0.17]. A significant interaction (*F* (1, 372) = 6.67, *p* = .010, η_p_^2^ = .02, *CI*_95%_ [0.00, 0.05]) suggested that making false‐positive errors damaged perceived warmth (*M* = 3.7, *SD* = 1.0) the most, compared with other leaders. Conspiracy leaders (*M* = 4.9, *SD* = 1.2) were rated as slightly more competent than neutral leaders (*M* = 4.7, *SD* = 1.3) overall, *F* (1, 372) = 4.12, *p* = .043, η_p_^2^ = .01, *CI*_95%_ [0.00, 0.04], with no significant interaction (*p* = .670). These findings suggested that being falsely positive in conspiracy detection led to a loss of warmth but maintained a baseline perception of the leader's competence. The results of the ANOVAs are displayed in Figure 3, and the detailed statistics of perceived warmth and competence were presented in the OSM.

### Discussion

Study 1 found that (1) conspiracy leaders were perceived as more dominant and less warm compared to neutral leaders, supporting H1.1 (Cao et al., 2025); (2) false‐negative leaders were perceived as the least dominant among all leaders, and no difference in dominance was found among correct leaders; (3) correct leaders, compared to false leaders, were generally perceived as more dominant, competent and warmer, supporting H1.2; and (4) conspiracy leaders were perceived as warmer when their theories were correct, partially supporting H1.3.

Study 1 suggests that the only condition that diminishes a leader's perceived dominance is a false‐negative error, which supports our theory that disseminating conspiracy theories can be a beneficial strategy for acquiring a reputation as a strong leader during intergroup conflict. Additionally, when conspiracy leaders were correct (vs. false), they were perceived as warmer, whereas neutral leaders' warmth (although consistently perceived as warmer), was not influenced by their correctness. Previous research indicates that leaders who exhibit dominance are often favoured during intergroup conflict (Laustsen & Petersen, 2017). To gain support and mobilize followers, leaders may strive to project an image of dominance, and spreading conspiracy theories can be instrumental to this purpose.

## STUDY 2

Study 1 found that leaders were perceived as more dominant but less warm when they erred on the side of conspiracy thinking (i.e., false positives). The results supported our theory that leaders' reputation may benefit from false positives rather than false negatives (i.e., dismissing conspiracy theories) in intergroup conflict. However, it is not clear whether this effect remains when there is no salient conflict. To further test our line of reasoning, Study 2 therefore manipulated error types (false positive vs. false negative) and the presence of intergroup conflict (present vs. absent).

In Study 1, intergroup conflict was present in all conditions, and false‐negative leaders were perceived as less dominant than false‐positive leaders, possibly because the former failed to address threats to their group. However, the reduction in dominance might be less pronounced in a situation without conflict. Therefore, we hypothesized that the difference in perceived dominance between leaders making false‐positives versus false‐negatives is smaller in the absence as opposed to the presence of conflict.

Furthermore, in the absence of conflict, a false positive may not be seen as a rational response and could harm intergroup communication and cooperation. Instead, leaders making a false negative may be viewed as more competent and warmer because this strategy may be more rational in conflict‐free situations. When conflict is present, however, false positives align with the expectation that leaders take possible threats into account, and false‐negative preferences may be perceived as maladaptive. As a result, false‐negative leaders' perceived competence may decrease when intergroup conflict is present as compared to when it is absent.

In summary, we hypothesized that: (1) leaders who make false positives are seen as more dominant but less warm than those who make false negatives (H2.1); (2) the perceived dominance of false‐positive leaders compared to false‐negative leaders are more pronounced when conflict is present as opposed to absent (H2.2); and (3) compared to conflict‐present situations, false‐negative leaders will be perceived as more competent in conflict‐absent situations (H2.3).

### Method

#### Participants

A minimum of 351 participants is required to achieve 80% power (with a small‐to‐medium‐sized effect for the interaction, effect size *f* = .15, α = .05). We recruited 400 participants (100 for each condition) from the United States via the Prolific platform. Those who failed the attention checks and the manipulation check were excluded, resulting in 331 eligible participants (161 males, 167 females and 3 others, ranging from 19 to 78 years old, *M* = 43.1, *SD* = 13.7). A sensitivity analysis via G*power showed that the minimum effect size of an interaction that can be reliably detected with this sample size is *f* = 0.154 (critical *F*(327) = 1.20, 80% power, α = .05).

#### Materials

The study had a 2 (false positive vs. false negative) by 2 (conflict‐present vs. conflict‐absent) design and was based on the same setting as Study 1. There was a conflict‐present condition, as in Study 1, where two tribes were in constant competition for food and a conflict‐absent condition where the tribes ‘have no communication or conflicts with each other’. The leader, Aru, had a different wrong perception depending on the condition: (1) Aru believed that the snakes were sent by their enemy, but it was later revealed to be an accident (the false positive condition); or (2) Aru assumed that the snakes appeared by accident, but it was later discovered to be a conspiracy (the false negative condition). The detailed materials are in the OSM.

#### Measurements

The same measurements as Study 1 were used, and internal reliabilities were good (α_warmth_ = .94, α_competence_ = .83, α_dominance_ = .91).

#### Procedure

Participants were randomly assigned to one of the four conditions. After reading the scenario, participants responded to the trait‐impression questions.

### Results

#### Descriptive results

The means, standard deviations and correlations are shown in Table 2.

**TABLE 2:** The means, standard deviations and correlations of measured variables (*N* = 331).

	*M*	*SD*	1	2	3
1. Age	43.1	13.7	‐		
2. Dominance	4.7	1.1	.05	‐	
3. Competence	4.0	1.0	.11	.52***	‐
4. Warmth	4.1	1.2	.13*	.03	.45***

*Note*: *p < .05; ***p < .001.

#### Hypothesis testing

Participants perceived false‐positive leaders as more dominant (*M* = 5.0, *SD* = 1.0) than false‐negative leaders (*M* = 4.4, *SD* = 1.2; H2.1 was supported), *F* (1, 327) = 26.08, *p* < .001, η_p_^2^ = .07, *CI*_95%_ [0.03, 0.13]. No main effect of condition was found, *F* (1, 327) = 0.40, *p* = .530, η_p_^2^ < .01, *CI*_95%_ [0.00, 0.02]. A significant interaction was found, *F* (1, 327) = 4.96, *p* = .027, η_p_^2^ = .01, *CI*_95%_ [0.00, 0.05]. Multiple comparisons suggested that, during intergroup conflict, leaders making false‐positive claims were considered more dominant (*M* = 5.1, *SD* = 1.1) than those making false‐negative claims (*M* = 4.2, *SD* = 1.2), *t*(327) = −5.16, *p* < .001. However, we did not find the difference between false‐positive (*M* = 4.9, *SD* = 1.1) and false‐negative (*M* = 4.6, *SD* = 1.0) leaders in the conflict‐absent condition, *t*(327) = −2.10, *p* = .218, which supported H2.2. These results replicated the findings in Study 1, suggesting that false positives produced a more dominant reputation, especially during intergroup conflict.

**FIGURE 4:**
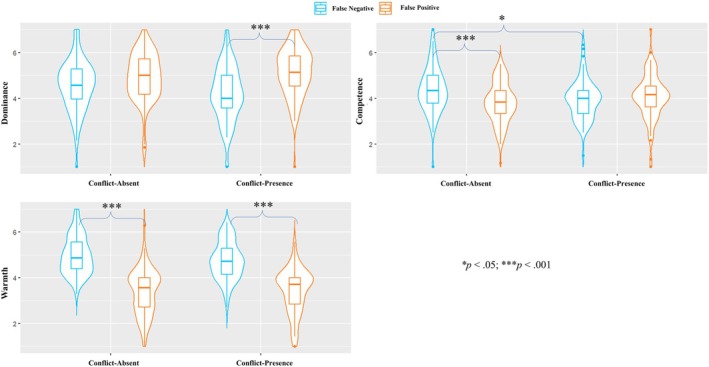
The results of the two‐way ANOVAs (Bonferroni corrected).

False‐positive leaders were perceived as less warm (*M* = 3.4, *SD* = 1.0) than false‐negative leaders (*M* = 4.9, *SD* = 0.8; H2.1 was supported), *F* (1, 327) = 210.24, *p* < .001, η_p_^2^ = .39, *CI*_95%_ [0.31, 0.46]. Neither the presence of intergroup conflict (*p* = .408) nor its interaction with error type (*p* = .161) significantly altered this decrease. Conversely, perceived competence yielded a significant interaction, *F* (1, 327) = 11.37, *p* < .001, η_p_^2^ = .03, *CI*_95%_ [0.01, 0.08]. False‐negative leaders in the conflict‐absent condition (*M* = 4.4, *SD* = 1.0) were perceived as more competent than in the conflict‐present condition (*M* = 3.9, *SD* = 0.9), *t*(327) = 2.83, *p* = .030, supporting H2.3. In contrast, no difference was found between false‐positive leaders in the conflict‐present (*M* = 4.1, *SD* = 1.0) versus conflict‐absent conditions (*M* = 3.8, *SD* = 0.9), *t*(327) = −1.92, *p* = .336. The results indicated that while the perceived warmth of false‐positive leaders remained low, being false‐positive (vs. false‐negative) did not influence perceived competence during intergroup conflicts. It then allowed the leaders' perceived dominance to emerge as an influencing trait. The results of the ANOVAs are displayed in Figure 4.

### Discussion

Study 2 supported all hypotheses and replicated the findings of Study 1. This study suggested that making false positives does not reduce dominance and competence during intergroup conflict. However, Studies 1 and 2 utilized a tribal scenario, which is quite remote from the context of modern societies. To replicate the findings and increase the external validity of the present research, we conducted Study 3 in a contemporary business environment.

## STUDY 3

This study replaced the conflict‐absent condition with a cooperation condition. Since false positives were not preferred in conflict‐absent conditions, they are likely to also be devalued in cooperation conditions, where false positives could harm cooperation. Thus, this study employed a 2 (conflict vs. cooperation) * 2 (false positive vs. false negative) between‐subject design. The study was pre‐registered on the OSF (https://osf.io/gbczn/overview?view_only=b3ae852db44d4f908ac2a60f080aa859).

We hypothesized that: (1) leaders who make false positives are perceived as more dominant than those who make false negatives (H3.1); (2) in the conflict condition, leaders who make false positives are perceived as more dominant than those who make false negatives (H3.2); (3) leaders who make false positives are perceived as less warm than those who make false negatives (H3.3); (4) in the cooperation condition, leaders who make false negatives are perceived as more competent than those who make false positives (H3.4); and (5) leaders who make false negatives are perceived as less competent in conflict than in cooperation situations (H3.5).

### Method

#### Participants

A minimum of 351 participants was required to achieve 80% power (with a small‐to‐medium‐sized effect for the interaction, effect size *f* = .15, α = .05), according to G*power. We recruited 400 participants (100 for each condition) from the United States via Prolific. Those who failed the attention checks and the manipulation check were excluded, resulting in 352 eligible participants (172 males, 178 females and 2 others, ranging from 20 to 78 years old, *M* = 42.5, *SD* = 12.1).

#### Materials

In line with the scenario framework used in the previous studies, we applied a modern organizational context. Participants imagined themselves as employees in a company that was either in competition or cooperation with another company. A significant event occurred where the company's IT system failed just hours before a crucial commercial event, resulting in substantial profit losses. The leaders of the department claimed that (1) the breakdown was due to a cyber‐attack from the competing company, but it was later revealed to be a system bug, which represented a false positive; or (2) the breakdown was due to a system bug, but it was later discovered to be a cyber‐attack from the competing company, which represented a false negative. The detailed materials are in the OSM.

#### Measurements

The same measurements as Study 1 were used, whose internal reliabilities were good (α_warmth_ = .93, α_competence_ = .85, α_dominance_ = .91).

#### Procedure

Participants were randomly assigned to one of the four conditions. After reading the scenario, participants responded to the trait‐impression questions.

### Results

#### Descriptive results

The means, standard deviations and correlations are shown in Table 3.

**TABLE 3:** The means, standard deviations and correlations of measured variables (*N* = 352).

	*M*	*SD*	1	2	3
1. Age	42.5	12.1	‐		
2. Dominance	4.6	1.1	.12*	‐	
3. Competence	3.9	1.0	.12*	.39***	‐
4. Warmth	3.8	1.1	.10	.02	.61***

*Note*: *p < .05; ***p < .001.

#### Hypothesis testing

People considered false‐positive leaders as more dominant (*M* = 4.9, *SD* = 1.1) than false‐negative leaders (*M* = 4.3, *SD* = 1.1; H3.1 was supported), *F* (1, 348) = 27.01, *p* < .001, η_p_^2^ = .07, *CI*_95%_ [0.03, 0.13]. No main effect of condition was found, *F* (1, 348) = 0.18, *p* = .676, η_p_^2^ < .01, *CI*_95%_ [0.00, 0.02]. A significant interaction was found, *F* (1, 348) = 4.65, *p* = .032, η_p_^2^ = .01, *CI*_95%_ [0.00, 0.05]. Multiple comparisons suggested that, during conflict, leaders making false‐positive claims were considered more dominant (*M* = 5.0, *SD* = 1.1) than those making false‐negative claims (*M* = 4.2, *SD* = 1.1; H3.2 was supported), *t*(348) = − 5.24, *p* < .001. However, we did not find the difference between false‐positive (*M* = 4.7, *SD* = 1.0) and false‐negative (*M* = 4.4, *SD* = 1.0) leaders in the cooperation condition, *t*(348) = − 2.08, *p* = .228. These results replicated the findings in Studies 1 and 2.

**FIGURE 5:**
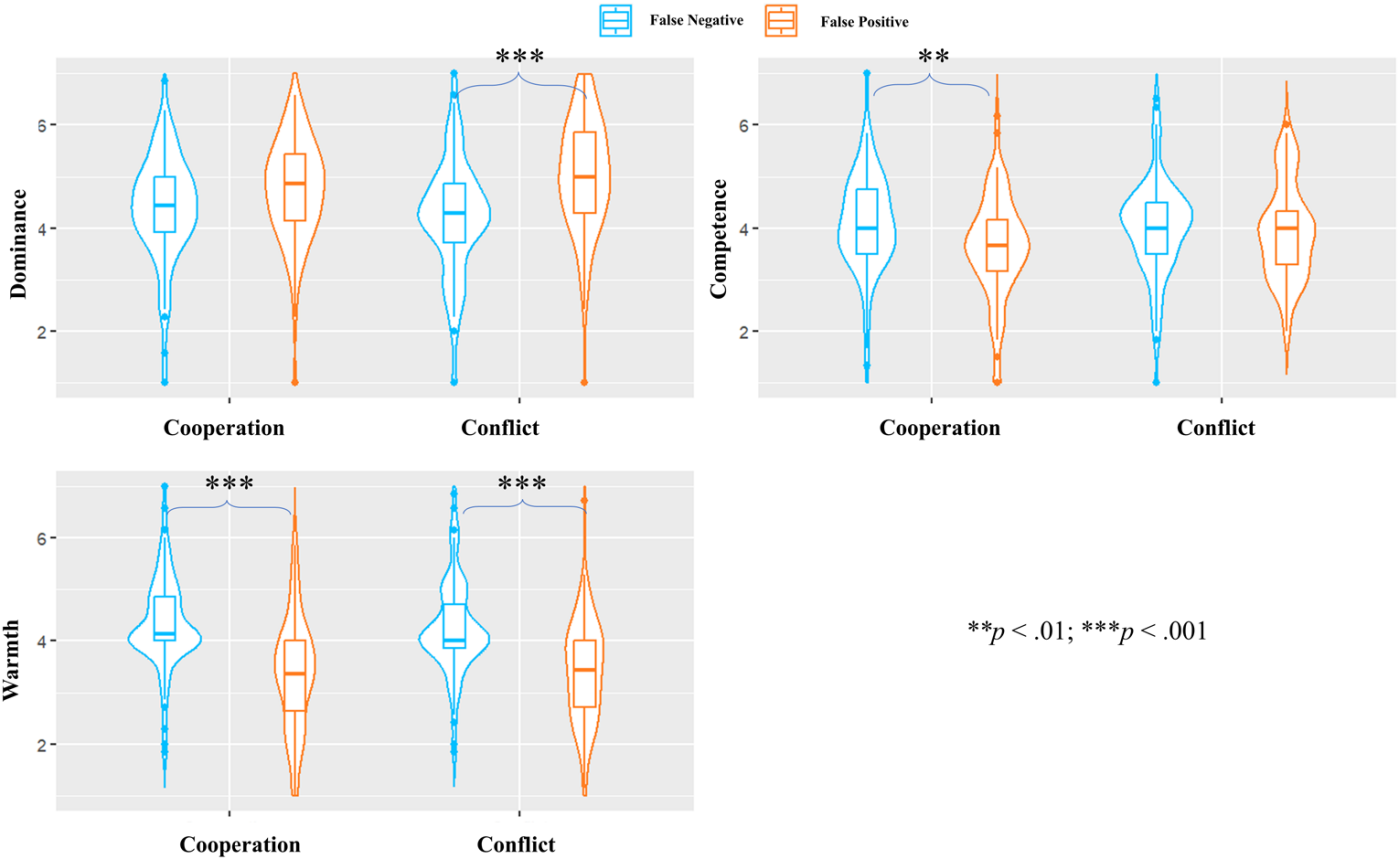
The results of the two‐way ANOVAs (Bonferroni corrected).

In line with previous studies, false‐positive leaders were perceived as less warm (*M* = 3.4, *SD* = 1.0) than false‐negative leaders (*M* = 4.3, *SD* = 0.9; H3.3 was supported), *F* (1, 348) = 77.34, *p* < .001, η_p_^2^ = .18, *CI*_95%_ [0.11, 0.25]. This effect remained across contexts, showing neither a main effect of the conflict condition (*p* = .886) nor a significant interaction (*p* = .457). In contrast, perceived competence was sensitive to the conflict context, yielding a significant interaction, *F* (1, 348) = 4.16, *p* = .042, η_p_^2^ = .01, *CI*_95%_ [0.00, 0.04]. In the cooperation condition, false‐positive leaders (*M* = 3.6, *SD* = 1.0) were perceived as less competent than false‐negative leaders (*M* = 4.1, *SD* = 0.9; H3.4 was supported), *t*(348) = 3.44, *p* = .004. However, in the conflict condition, such a difference was not found (*M*_positive_ = 3.9, *SD*_positive_ = 0.9; *M*_negative_ = 4.0, *SD*_negative_ = 0.9), *t*(348) = 0.61, *p* = 1.000. Although no difference emerged between false‐negative leaders in the cooperation condition and in the conflict condition, *t*(348) = 0.66, *p* = 1.000 (H3.5 was not supported), the structural outcome remained identical: the perceived competence gap between a false positive and false negative disappeared in a conflict setting. The results of ANOVAs are displayed in Figure 5.

### Discussion

Study 3 supported our hypotheses except for H3.5, replicating most of the findings from Study 2. The cumulative evidence from the first three studies implied that during intergroup conflict, false‐positive errors increase the extent to which leaders are seen as dominant without influencing their perceived competence. In the context of intergroup cooperation, in contrast, perceived dominance for both types of leaders is similar. Instead, false‐positive errors can significantly decrease their perceived competence. Regardless of the situation, leaders who make false‐positive errors are perceived as less warm than those who make false‐negative errors. These findings highlight a potential trade‐off in leaders' reputation depending on whether the context is one of conflict or cooperation.

## STUDY 4

In our previous studies, we included three different conditions: cooperation, conflict‐absent and conflict‐present. Each context could be associated with different costs when potential conspiracies are overlooked or when another group is falsely accused of conspiring. We found that false positives and false negatives with various costs influenced leaders' reputations differently.

Thus, Study 4 directly manipulated the cost (high vs. low) of false negatives and tested how leaders (false‐positive leaders vs. false‐negative leaders) were perceived by their followers. We employed a scenario of modern warfare where two parties were fighting for dominance of a country. In the high‐cost condition, a false negative resulted in losing the conflict and being deported, while in the low‐cost condition, a false negative did not influence the war. This study was pre‐registered on the OSF (https://osf.io/8rjzk/overview?view_only=ae13f333d279413ea56544a86f7b1b4c).

We hypothesized that: (1) false‐positive leaders are perceived as more dominant than false‐negative leaders (H4.1); (2) in the low‐cost condition, both leaders are perceived as similarly dominant (H4.2); (3) in the high‐cost condition, false‐positive leaders are perceived as more dominant than false‐negative leaders (H4.3); (4) false‐negative leaders are perceived as warmer than false‐positive leaders (H4.4); (5) in the low‐cost condition, both leaders are perceived as similarly competent (H4.5); and (6) in the high‐cost condition, false‐positive leaders are perceived as more competent than false‐negative leaders (H4.6).

### Method

#### Participants

A minimum of 351 participants were required to achieve 80% power (with a small‐to‐medium‐sized effect for the interaction, effect size *f* = .15, α = .05). We recruited 400 participants (100 for each condition) from the United States via the Prolific. Those who failed the attention checks and the manipulation check were excluded, resulting in 378 eligible participants (187 males, 185 females and 6 others, ranging from 18 to 81 years old, *M* = 38.4, *SD* = 11.7).

#### Materials

We adjusted the scenarios to fit a wartime context. Participants imagined that they were members of a political faction in an African country, engaged in conflict with another faction. A fire broke out at their military storage facility, leading to a large explosion and numerous casualties. In the high‐cost condition, their faction had a limited supply of ammunition. If similar incidents were to occur at other military facilities, their faction could potentially lose the conflict and face the risk of deportation. In contrast, in the low‐cost condition, their faction had an ample supply of ammunition. Therefore, a similar incident would not significantly impact them. In the false positive condition, the faction leader claimed that the fire was set by their enemy. However, participants read at the end of the materials that the true reason was ‘the escalating temperature and dry conditions’ (false positive). In the false negative condition, the faction leader claimed that the fire occurred due to ‘the escalating temperature and dry conditions’, but participants later read that it was set by their enemy (false negative). The detailed materials are in the OSM.

#### Measurements

The same measurements as Study 1 were used, and again internal reliabilities were good (α_warmth_ = .92, α_competence_ = .83, α_dominance_ = .90).

#### Procedure

Participants were randomly assigned to one of the four conditions. After reading the scenario, participants responded to the trait‐impression questions.

### Results

#### Descriptive results

The means, standard deviations and correlations are shown in Table 4.

**TABLE 4:** The means, standard deviations and correlations of measured variables (*N* = 378).

	*M*	*SD*	1	2	3
1. Age	38.4	11.7	‐		
2. Dominance	4.5	1.1	.03	‐	
3. Competence	3.8	0.9	.08	.48***	‐
4. Warmth	3.7	1.1	.09	−.11*	.46***

*Note*: *p < .05; ***p < .001.

#### Hypothesis testing

Participants considered false‐positive leaders as more dominant (*M* = 4.8, *SD* = 1.0) than false‐negative leaders (*M* = 4.1, *SD* = 1.1; H4.1 was supported), *F* (1, 374) = 46.43, *p* < .001, η_p_^2^ = .11, *CI*_95%_ [0.06, 0.17]. A significant interaction was found, *F* (1, 374) = 4.91, *p* = .027, η_p_^2^ = .01, *CI*_95%_ [0.00, 0.04]. Multiple comparisons suggested that the difference between false‐positive and false‐negative leaders was bigger in the high‐cost condition (*M*_positive_ = 5.0, *SD*_positive_ = 0.8; *M*_negative_ = 4.1, *SD*_negative_ = 1.1; difference = 0.9, *t*(372) = −6.45, *p* < .001; H4.3 was supported), compared to the low‐cost condition (*M*_positive_ = 4.7, *SD*_positive_ = 1.1; *M*_negative_ = 4.2, *SD*_negative_ = 1.1; difference = 0.5, *t*(372) = −3.14, *p* = .011; H4.2 was not supported).

**FIGURE 6:**
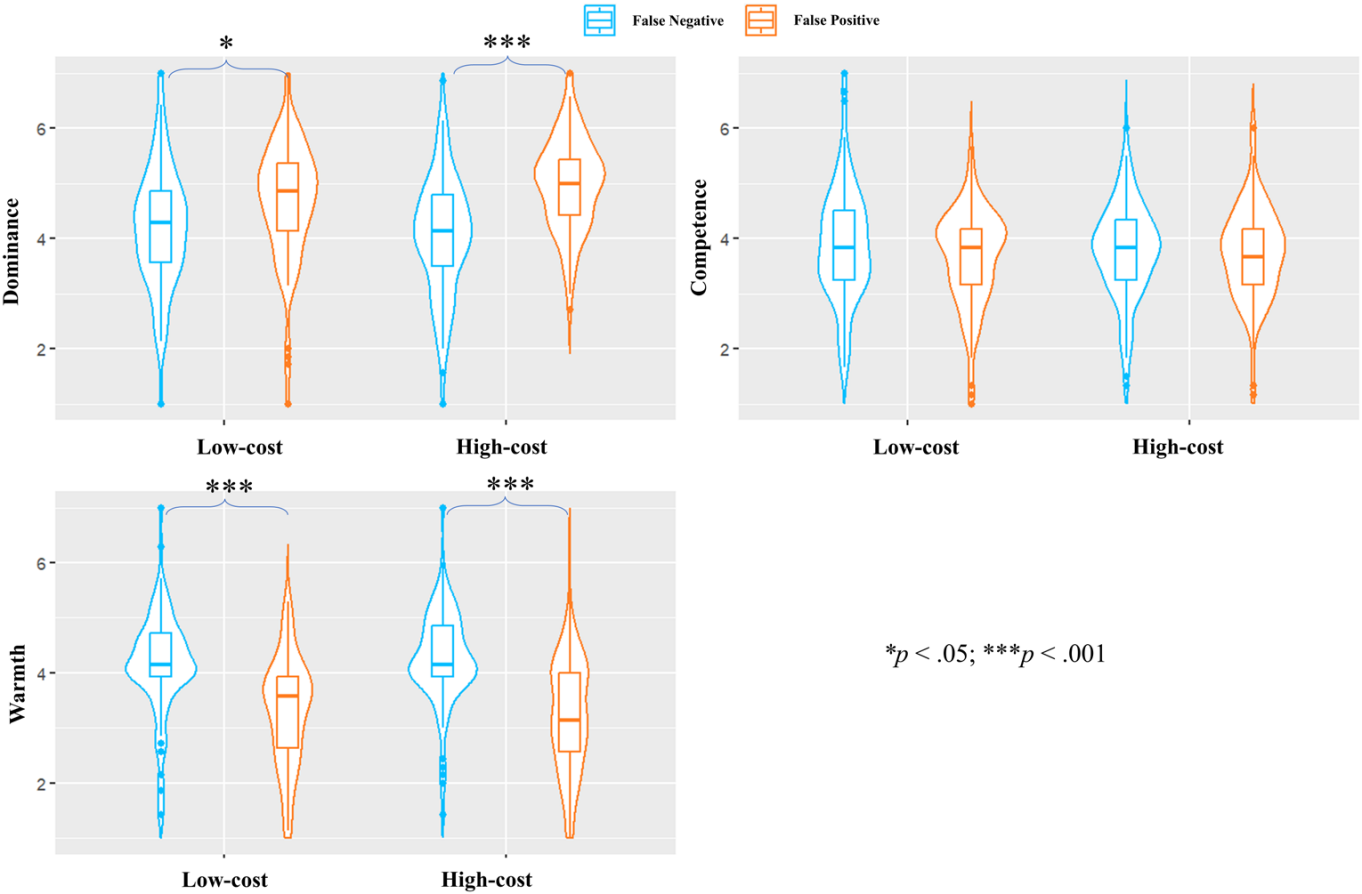
The results of the two‐way ANOVAs (Bonferroni corrected).

In line with previous findings, false‐negative leaders were perceived as warmer (*M* = 4.2, *SD* = 0.9) than false‐positive leaders (*M* = 3.3, *SD* = 1.0; H4.4 was supported), *F* (1, 374) = 95.25, *p* < .001, η_p_^2^ = .20, *CI*_95%_ [0.14, 0.27]. This effect was independent of conditions, showing neither a main effect of cost (*p* = .909) nor a significant interaction (*p* = .289). For perceived competence, no main effect of error type (*p* = .138), no main effect of cost (*p* = .813) and no interaction between the two (*p* = .269; H4.5 and H4.6 were not supported). The results of the ANOVAs are displayed in Figure 6.

### Discussion

Study 4 revealed that leaders who make false positives were perceived as more dominant than those who make false negatives. This difference in perception was amplified when the costs of overlooking potential conspiracies were high compared to low. Costs of false negatives did not significantly influence perceived competence. While not all our hypotheses were supported, the results were broadly consistent with the theory that the costs of errors in perceiving conspiracies (i.e., false positives versus false negatives) might differentially influence leaders' reputations.

## GENERAL DISCUSSION

### Overview

Grounded in EMT and the evolutionary dynamics of leader–follower relations, this research examined how false‐positive and false‐negative errors in conspiracy detection affect leaders' reputations. Across four studies, we consistently observed a dominance‐warmth trade‐off contingent upon various contexts. In Study 1, we found that false‐negative leaders were perceived as the least dominant, while false‐positive, accurate (hits) and correct‐rejection leaders were viewed similarly dominant during intergroup conflict. Studies 2 and 3 replicated and extended these findings to diverse scenarios (conflict‐present, conflict‐absent and cooperative contexts) using hunter‐gatherer and modern settings. In conflict conditions, false‐positive leaders were perceived as more dominant than false‐negative leaders and similarly competent. However, in conflict‐absent settings, false‐positive leaders were viewed as similarly dominant but less competent than false‐negative leaders. Across all contexts, false‐positive leaders were consistently perceived as less warm.

Study 4 expanded on these findings by manipulating the cost of false negatives in a modern war setting. Results showed that false‐positive leaders were perceived as more dominant than false‐negative leaders, with this dominance gap widening as the cost of false negatives increased. These findings align with prior research (Cao et al., 2025; Green, Toribio‐Flórez, & Douglas, 2023; Green, Toribio‐Flórez, Douglas, Brunkow, & Sutton, 2023) and suggest that spreading conspiracy theories, even if false, functions as a costly signal that successfully bolsters perceived dominance, albeit at the expense of perceived warmth.

### Implications to leadership perceptions

Our findings make a novel contribution to the psychology of leadership perception by unravelling the functional mechanism through which a leader's ability to detect conspiracies enhances their perceived dominance during intergroup conflict. Dominance, associated with high social rank, is particularly valued in conflict scenarios (Cheng et al., 2013; Little et al., 2007; van Vugt & Grabo, 2015) because it serves followers' evolutionary need for protection (Sheng et al., 2026). As the costs of false negatives rise, the recognition of leaders' dominance appears to intensify, suggesting an adaptive response to perceived existential threats. In conflict‐absent settings, however, conspiracy detection loses its strategic advantage, resulting in less favourable perceptions of false‐positive leaders. Ultimately, intergroup conflict seems to amplify followers' need for protection, allowing them to attribute dominance to hyper‐vigilant leaders.

Our findings supported the proposed dominance‐warmth trade‐off. Perceivers are ‘motivated tacticians who consider social interaction goals’ (Stevens & Fiske, 1995, p. 189) who dynamically shift their trait preferences based on contextual demands. As Sheng et al. (2026) stated, while the need for leader competence remains stable across contexts, the specific need for affiliation (warmth) is prioritized during intragroup cooperation, whereas the need for protection (dominance) is adaptive when facing intergroup threats. Consequently, although warmth and competence are valued, the utility of dominant leaders rises when intergroup conflicts are salient (van Vugt & Smith, 2019). Within this functional framework, the hyper‐vigilance of a false‐positive leader aligns with follower expectations during conflict, satisfying the acute demand for a dominant protector, in which the decreased warmth appears to be less important. Conversely, in conflict‐absent contexts, this paranoid behaviour contradicts the need for cooperative warmth, leading to negative evaluations (Lord et al., 2020).

### Implications for leadership strategy

Our studies suggest that spreading conspiracy theories allows leaders to project dominance as a strategy, particularly in conflict settings (Halevy et al., 2012; Spisak et al., 2015). This strategy may resonate with conservative audiences who prioritize strength over warmth in leadership (Laustsen, 2017). For instance, leaders like Donald Trump have successfully leveraged conspiracy theories to appeal to conservative supporters by projecting toughness and decisiveness. However, this approach may carry significant reputational risks and outcomes in conflict‐absent situations, where warmth and competence are valued more highly. This interpretation was supported by an exploratory analysis where we examined whether participants' impressions were related to their leadership preferences using a moderated mediation model (see the OSM for detailed results). When conflict was absent or cooperation was required, false‐positive leaders were evaluated as having lower general reputation (i.e., warmth, competence and dominance), which further related to lower leadership preference. By contrast, when conflict was present, the preference for false‐positive (vs. false‐negative) leaders was similar and not related to the leader's reputation. These results implied that being a false‐positive leader during conflict (vs. conflict‐absent), carried lower reputational costs and might not necessarily be associated with decreased leadership preference.

### Contributions, limitations and future directions

This research makes two key novel contributions. First, it empirically examines, from an error‐management perspective and fundamental followers' needs, how leaders' conspiracy detection abilities impact their reputations in both conflict and conflict‐absent contexts. The findings suggest that spreading conspiracy theories can serve as an adaptive error management strategy, particularly during intergroup conflict when the cost of false negatives is high. Second, the research provides novel evidence that excessive vigilance to conspiracies (false positives) enhances perceived dominance in conflict scenarios. Therefore, the results suggest an intriguing implication: in situations of intergroup conflict where the likelihood of conspiracies is uncertain, dominant leaders might find it more beneficial to propagate conspiracy theories rather than provide neutral explanations if they wish to maintain their dominant image.

Despite its contributions, this research has limitations. The studies relied on scenario‐based methods, where participants read descriptions of leaders. While common in impression formation research (Brambilla et al., 2012; van der Zanden et al., 2020), future studies could use behavioural experiments or field studies to observe real‐world implications, such as voting behaviour. Additionally, it remains unclear under what conditions false‐positive leaders might surpass false‐negative leaders in competence perceptions during intergroup conflict. Future work could explore this question and examine more nuanced traits, such as trustworthiness or political impact, to better understand the reputational dynamics of conspiracy‐spreading leaders.

Moreover, the current research did not explicitly capture prestige‐based status. Grounded in the service‐for‐prestige theory (Price & van Vugt, 2014), leadership status is conferred by followers in exchange for group services. Future studies may examine whether followers offer a prestigious reputation for the conspiracy detection service. Furthermore, the present studies did not manipulate whether the target leaders were the ones that participants supported. Future research may investigate follower‐leader congruence (e.g., supported vs. opposed leaders) or partisanship (e.g., Cassidy et al., 2022; Clifford, 2019) as a boundary condition to further delineate for whom and when these reputational trade‐offs emerge.

### CONCLUSION

These studies reveal that during intergroup conflict, spreading conspiracy theories, even when proven false, does not greatly damage leaders' reputations. Instead, false‐positive leaders are perceived as less warm but as similarly competent and even more dominant than false‐negative leaders. The dominance gap widens as the cost of false‐negative errors increases. In conflict‐absent conditions, however, false‐positive leaders are viewed as less competent and warm than false‐negative leaders.

From an error management perspective, these findings suggest that spreading conspiracy theories can be an effective strategy for leaders to foster a reputation of strength and dominance, particularly during conflict. This research highlights the trade‐offs involved in such strategies, offering valuable insights into the psychological and contextual factors shaping the reputation of leaders.

## AUTHOR CONTRIBUTIONS

**Shen Cao:** Conceptualization; methodology; software; investigation; validation; formal analysis; funding acquisition; visualization; writing – original draft; writing – review and editing. **Jan‐Willem van Prooijen:** Conceptualization; methodology; validation; supervision; writing – review and editing. **Mark van Vugt:** Conceptualization; methodology; validation; supervision; writing – review and editing.

## CONFLICT OF INTEREST STATEMENT

The authors declare no conflict of interests.

## Supporting information


**Data S1.** Supporting Information.

## Data Availability

The data that support the findings of this study are openly available in the Open Science Framework (https://osf.io/r2wx4/overview?view_only=4bb92b7a7a884fa98e7253bf66f1883a).
