# Supplementary material for: Sharing conspiracy theories and staying in power: How leaders' false theories influence leadership perception
Source: Br J Soc Psychol. 2026 Apr 28;65:e70088. doi: 10.1111/bjso.70088 (PMC13125733; doi:10.1111/bjso.70088)
Supplement: Supplementary file 1 — Data S1. Supporting Information. [file BJSO-65-0-s001.zip › Detailed results of warmth & competence.docx]

**Study 1**

For perceived warmth, leaders making correct inferences were perceived as warmer (*M* = 4.7, *SD* = 0.9) than those making false inferences (*M* = 4.1, *SD* = 1.0), *F* (1, 372) = 39.41, *p* < .001, η_p_^2^ = .10, *CI*_95%_ [0.05, 0.16]. Moreover, people considered conspiracy leaders as less warm (*M* = 4.1, *SD* = 1.0) than the neutral ones (*M* = 4.7, *SD* = 0.9), *F* (1, 372) = 47.01, *p* < .001, η_p_^2^ = .11, *CI*_95%_ [0.06, 0.17]. A significant interaction also emerged, *F* (1, 372) = 6.67, *p* = .010, η_p_^2^ = .02, *CI*_95%_ [0.00, 0.05]. Multiple comparisons with Bonferroni adjustment suggested that leaders making false-positive claims were considered as the least warm (*M* = 3.7, *SD* = 1.0) than other leaders. Furthermore, the difference between neutral and conspiracy leaders was bigger when they made errors (*M*_neutral_ = 4.5, *SD*_neutral_ = 0.8; *M*_conspiracy_ = 3.7, *SD*_conspiracy_ = 1.0; difference = 0.9, *t*(372) = 6.63, *p* < .001), than when the leaders were correct (*M*_neutral_ = 4.9, *SD*_neutral_ = 0.9; *M*_conspiracy_ = 4.5, *SD*_conspiracy_ = 0.9; difference = 0.4, *t*(372) = 3.13, *p* = .011). In addition, the difference between correct and false leaders was bigger in the conspiracy condition (difference = 0.8, *t*(372) = 6.33, *p* < .001), compared to the neutral condition (difference = 0.3, *t*(372) = 2.63, *p* = .053).

For perceived competence, leaders making correct inferences were perceived as more competent (*M* = 5.7, *SD* = 0.8) than those making false inferences (*M* = 4.0, *SD* = 1.0), *F* (1, 372) = 320.28, *p* < .001, η_p_^2^ = .46, *CI*_95%_ [0.39, 0.52]. Moreover, people considered leaders sharing conspiracy theories as slightly more competent (*M* = 4.9, *SD* = 1.2) than the neutral ones (*M* = 4.7, *SD* = 1.3), *F* (1, 372) = 4.12, *p* = .043, η_p_^2^ = .01, *CI*_95%_ [0.00, 0.04]. No significant interaction was found, *F* (1, 372) = 0.18, *p* = .670, η_p_^2^ < .01, *CI*_95%_ [0.00, 0.01].

**Study 2**

False-negative leaders were perceived as warmer (*M* = 4.9, *SD* = 0.8) than false-positive leaders (*M* = 3.4, *SD* = 1.0; H2.1 was supported), *F* (1, 327) = 210.24, *p* < .001, η_p_^2^ = .39, *CI*_95%_ [0.31, 0.46]. Results indicated neither a main effect of conflict, *F* (1, 327) = 0.69, *p* = .408, η_p_^2^ < .01, *CI*_95%_ [0.00, 0.02], nor an interaction, *F* (1, 327) = 1.98, *p* = .161, η_p_^2^ < .01, *CI*_95%_ [0.00, 0.03].

False-positive leaders (*M* = 3.9, *SD* = 0.9) were perceived as less competent than false-negative leaders (*M* = 4.2, *SD* = 1.0), *F* (1, 327) = 4.98, *p* = .026, η_p_^2^ = .02, *CI*_95%_ [0.00, 0.05]. No main effect of condition was found, *F* (1, 327) = 0.31, *p* = .578, η_p_^2^ < .01, *CI*_95%_ [0.00, 0.02]. A significant interaction emerged, *F* (1, 327) = 11.37, *p* < .001, η_p_^2^ = .03, *CI*_95%_ [0.01, 0.08]. Multiple comparisons suggested that false-negative leaders in the conflict-absent condition (*M* = 4.4, *SD* = 1.0) were perceived as more competent than in the conflict-present condition (*M* = 3.9, *SD* = 0.9), *t*(327) = 2.83, *p* = .030, supporting H2.3. In contrast, no difference was found between false-positive leaders in the conflict-present (*M* = 4.1, *SD* = 1.0) versus conflict-absent conditions (*M* = 3.8, *SD* = 0.9), *t*(327) = -1.92, *p* = .336. Furthermore, in the conflict-absent condition, false-positive leaders were perceived as less competent than false-negative leaders, *t*(327) = 3.96, *p* < .001. However, in the conflict-present condition, such a difference was not found between the two leaders, *t*(327) = -0.86, *p* = 1.000.

**Study 3**

False-negative leaders were perceived as warmer (*M* = 4.3, *SD* = 0.9) than false-positive leaders (*M* = 3.4, *SD* = 1.0; H3.1 was supported), *F* (1, 348) = 77.34, *p* < .001, η_p_^2^ = .18, *CI*_95%_ [0.11, 0.25]. No main effect of conflict was found, *F* (1, 348) = 0.02, *p* = .886, η_p_^2^ < .01, *CI*_95%_ [0.00, 0.01]. No interaction was found either, *F* (1, 348) = 0.56, *p* = .457, η_p_^2^ < .01, *CI*_95%_ [0.00, 0.02].

Furthermore, false-positive leaders (*M* = 3.8, *SD* = 1.0) were perceived as less competent than false-negative leaders (*M* = 4.1, *SD* = 0.9), *F* (1, 348) = 8.24, *p* = .004, η_p_^2^ = .02, *CI*_95%_ [0.00, 0.06]. No main effect of condition was found, *F* (1, 348) = 1.24, *p* = .266, η_p_^2^ < .01, *CI*_95%_ [0.00, 0.03]. A significant interaction occurred, *F* (1, 348) = 4.16, *p* = .042, η_p_^2^ = .01, *CI*_95%_ [0.00, 0.04]. Multiple comparisons suggested that, in the cooperation condition, false-positive leaders (*M* = 3.6, *SD* = 1.0) were perceived as less competent than false-negative leaders (*M* = 4.1, *SD* = 0.9; H3.2 was supported), *t*(348) = 3.44, *p* =.004. However, in the conflict condition, such a difference was not found between the two leaders (*M*_positive_ = 3.9, *SD*_positive_ = 0.9; *M*_negative_ = 4.0, *SD*_negative_ = 0.9), *t*(348) = 0.61, *p* = 1.000. Different from Study 2, no difference emerged between false-negative leaders in the cooperation condition and in the conflict condition, *t*(348) = 0.66, *p* = 1.000 (H3.3 was not supported). No difference emerged between false-positive leaders in the conflict and cooperation conditions either, *t*(348) = -2.23, *p* = .159.

**Study 4**

False-negative leaders were perceived as warmer (*M* = 4.2, *SD* = 0.9) than false-positive leaders (*M* = 3.3, *SD* = 1.0; H4.1 was supported), *F* (1, 374) = 95.25, *p* < .001, η_p_^2^ = .20, *CI*_95%_ [0.14, 0.27]. No main effect of cost was found, *F* (1, 374) = 0.01, *p* = .909, η_p_^2^ < .01, *CI*_95%_ [0.00, 0.01]. No interaction was found, *F* (1, 374) = 1.13, *p* = .289, η_p_^2^ < .01, *CI*_95%_ [0.00, 0.02]. For perceived competence, no main effect of error type was found, *F* (1, 374) = 2.21, *p* = .138, η_p_^2^ < .01, *CI*_95%_ [0.00, 0.03], nor of cost, *F* (1, 374) = 0.06, *p* = .813, η_p_^2^ < .01, *CI*_95%_ [0.00, 0.01]. No interaction was found either, *F* (1, 374) = 1.23, *p* = .269, η_p_^2^ < .01, *CI*_95%_ [0.00, 0.02] (H4.2 and H4.3 were not supported).
