# Supplementary material for: Sharing conspiracy theories and staying in power: How leaders' false theories influence leadership perception
Source: Br J Soc Psychol. 2026 Apr 28;65:e70088. doi: 10.1111/bjso.70088 (PMC13125733; doi:10.1111/bjso.70088)
Supplement: Supplementary file 1 — Data S1. Supporting Information. [file BJSO-65-0-s001.zip › The results of an exploratory analysis.pdf]

Leader perception as a conditional mediator of leadership preference

In Studies 2-4, we measured leadership preference with a single item: "To what extent would you like Aru to be the leader of your tribe," following participants' assessments of perceived dominance, warmth, and competence. In an exploratory analysis, we examined whether participants' impressions were related to their leadership preferences using a moderated mediation model. Error type was treated as the independent variable (X), condition as the moderator, and warmth, competence, and dominance as parallel mediators (M_1–M_3). Leadership preference served as the dependent variable. Moderation was modeled on the paths from error type to each mediator (i.e., the X → M paths), allowing the indirect effects of error type on leadership preference to vary across levels of condition (absence vs. presence). No specific assumptions were made for this exploratory analysis.

The proposed model is as follows:

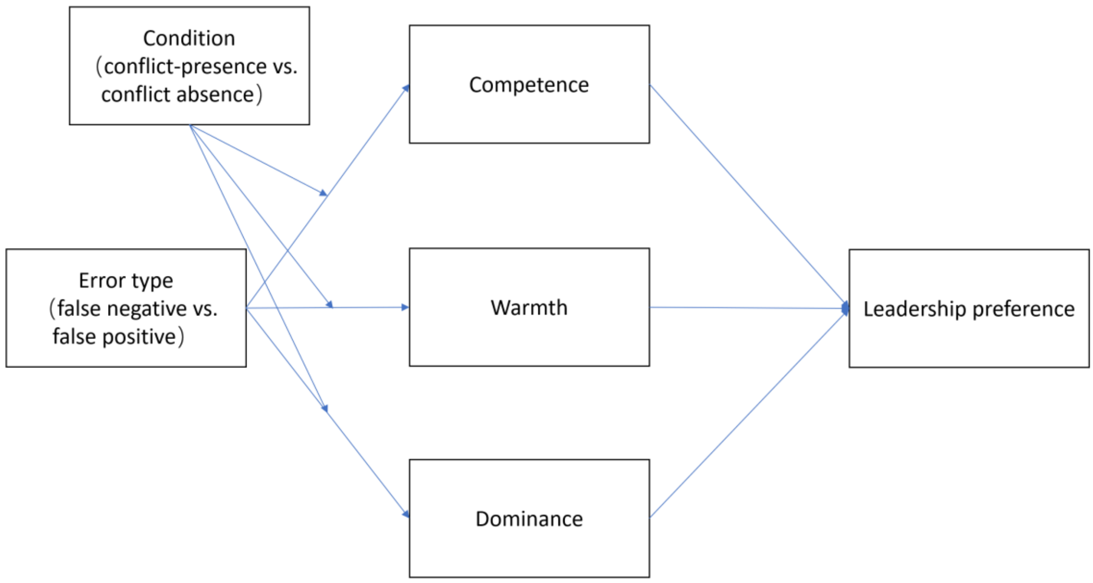

Data analysis

A structural equation model was estimated using maximum likelihood (ML) in R with the *lavaan* package (version 0.6-19). False positive (vs. false negative) served as the independent variable (IV). In Study 2, conflict-present (vs. conflict-absent) was the moderator. In Study 3, cooperation (vs. conflict) was the moderator, and in Study 4, high (vs. low) cost was the moderator. The full model was estimated based on Maximum Likelihood estimation with Bootstrap (n = 5000). Model fit including CFI, TLI, and RMSEA were reported.

Results

Study 2

Study 2 employed a 2 (error type: false positive vs. false negative) × 2 (conflict: present vs. absent) design in a tribal context. The model demonstrated an acceptable fit to the data, $\chi^2(1) = 5.40$, $p = .020$, with CFI = .994, TLI = .809, RMSEA = .115 (90% CI [.037, .218]), and SRMR = .006. The model accounted for 7.5% of the variance in competence, 40.5% in warmth, 9.7% in dominance, and 56.5% in leadership preference.

Error type ("0" = false negative; "1" = false positive) significantly predicted warmth ($b = -1.52$, p < .001), competence ($b = -0.61$, $p < .001$), and dominance ($b = 0.33$, $p = .047$), but not leadership preference ($b = -0.06$, $p = .725$). Condition ("0" = conflict-absent; "1" = conflict-present) significantly predicted dominance ($b = -0.37$, $p = .045$) and competence ($b = -0.42$, $p = .004$), but not warmth ($b = -0.23$, $p = .077$) and leadership preference ($b = -0.05$, $p = .662$). Leadership preference was significantly predicted by competence ($b = 0.98$, $p < .001$) and warmth ($b = 0.31$, $p = .001$), but not by dominance ($b = 0.09$, $p = .171$). A significant interaction between error type and

condition emerged for competence (*b* = 0.75, *p* < .001) and dominance (*b* = 0.57, *p* = .020), but not for warmth (*b* = 0.33, *p* = .095).

Significant conditional indirect effects were observed. The total indirect effect (from error type to leadership preference) in the conflict-absent condition (*b* = -1.08, 95% *CI* [-1.51, -0.63]) was larger than in the conflict-present condition (*b* = -0.19, 95% *CI* [-0.58, 0.22]), Δ*b* = 0.89, 95% *CI* [0.40, 1.40]. This suggests that in the conflict-absent condition, false-positive leaders were evaluated more negatively and associated with reduced willingness to select them as leaders. In contrast, in the conflict-present condition, willingness to choose false-positive leaders was not related to their decreased general reputation.

The difference in the total indirect effect was primarily driven by the indirect effect through perceived warmth and competence. Specifically, in the conflict-absent condition, participants perceived false-positive leaders (vs. false-negative leaders) as less competent (*b* = -0.61, 95% *CI* [-0.90, -0.32]), which further reduced their willingness to select those leaders (b_{ind} = -0.6, 95% *CI* [-0.91, -0.31]). In contrast, in the conflict-present condition, false-positive and false-negative leaders were perceived as similarly competent (*b* = 0.14, 95% *CI* [-0.14, 0.41]), resulting in no significant difference in leadership preference (b_{ind} = 0.14, 95% *CI* [-0.13, 0.41]). The indirect effect via warmth was comparable across conditions, with b_{ind} = -0.40, 95% *CI* [-0.65, -0.17] in the conflict-present condition and b_{ind} = -0.51, 95% *CI* [-0.82, -0.21] in the conflict-absent condition. No significant indirect effect was found through perceived dominance (b_{ind} = 0.08, 95% *CI* [-0.03, 0.22] in the conflict-present condition; b_{ind} = 0.03, 95% *CI* [-0.02, 0.09] in the conflict-absent condition). In sum, in the conflict-absent condition, preference for false-positive leaders decreased relative to false-negative leaders due to lower perceived competence and warmth,

whereas in the conflict-present condition, leadership preference did not differ because perceived competence was similar. Leadership preference was associated not only with warmth but with the combined effects of warmth and competence. The indirect effects are plotted in Figure 2.

Figure 2

Indirect effects (error type to leadership preference)

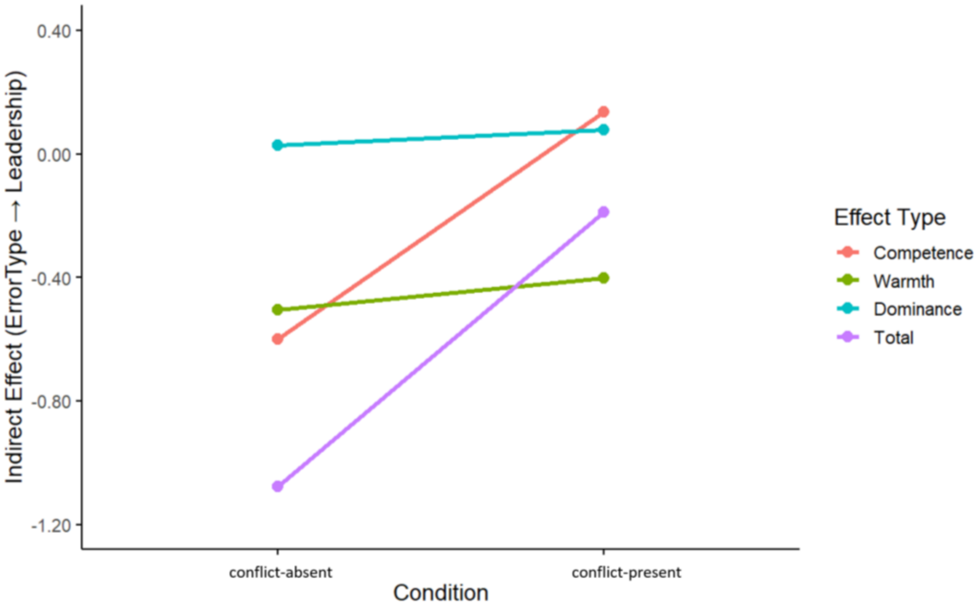

Study 3

Study 3 employed a 2 (error type: false positive vs. false negative) × 2 (condition: cooperation vs. conflict) design in an organizational context. The model demonstrated an acceptable fit to the data, $\chi^2(1) = 0.20$, $p = .657$, with $CFI = 1.000$, $TLI = 1.041$, $RMSEA < .001$ (90% CI [.000, .108]), and $SRMR = .001$. The model accounted for 4.2% of the variance in competence, 18.9% in warmth, 11.2% in dominance, and 47.7% in leadership preference.

Error type (“0” = false negative; “1” = false positive) significantly predicted warmth ($b = -0.99$, p < .001), competence ($b = -0.51$, p < .001), dominance ($b = 0.34$, p

= .033), and leadership preference (*b* = -0.31, *p* = .021). Different from Study 2, condition ("0" = cooperation; "1" = conflict) was not found predicting warmth (*b* = -0.08, *p* = .553), competence (*b* = -0.09, *p* = .530), or dominance (*b* = -0.25, *p* = .107), but predicted leadership preference (*b* = -0.31, *p* = .021). Leadership preference was significantly predicted by competence (*b* = 0.79, *p* < .001) and warmth (*b* = 0.24, *p* = .006), but not by dominance (*b* = -0.03, *p* = .658). A significant interaction term between error type and condition emerged for competence (*b* = 0.42, *p* = .042) and dominance (*b* = 0.54, *p* = .014), but not for warmth (*b* = 0.15, *p* = .459).

No significant conditional indirect effects were observed. The total indirect effect in the conflict-absent condition (*b* = -0.62, 95% *CI* [-0.92, -0.35]) was similar to that in the conflict-present condition (*b* = -0.28, 95% *CI* [-0.57, 0.01]), Δ*b* = 0.35, 95% *CI* [-0.04, 0.73].

Similar to Study 2, the difference in the total indirect effect was primarily driven by the indirect effect through perceived warmth and competence. Specifically, in the cooperation condition, the indirect effect through competence was significant (*b* = -0.40, 95% *CI* [-0.67, -0.17]). In contrast, in the conflict condition, this indirect effect was not significant (*b* = -0.07, 95% *CI* [-0.30, 0.14]), with a significant difference between conditions (Δ*b* = 0.33, 95% *CI* [0.02, 0.65]). For warmth, the indirect effects were significant in both cooperation (*b* = -0.21, 95% *CI* [-0.37, -0.06]) and conflict conditions (*b* = -0.18, 95% *CI* [-0.33, -0.05]), Δ*b* = 0.03, 95% *CI* [-0.06, 0.13]. The indirect effect via dominance was not significant in either the cooperation (*b* = -0.01, 95% *CI* [-0.07, 0.04]) or conflict condition (*b* = -0.03, 95% *CI* [-0.18, 0.10]), Δ*b* = -0.02, 95% *CI* [-0.13, 0.06]. In sum, the patterns of indirect effects were in line with Study 2, which are plotted in Figure 3.

Figure 3

Indirect effects (error type to leadership preference)

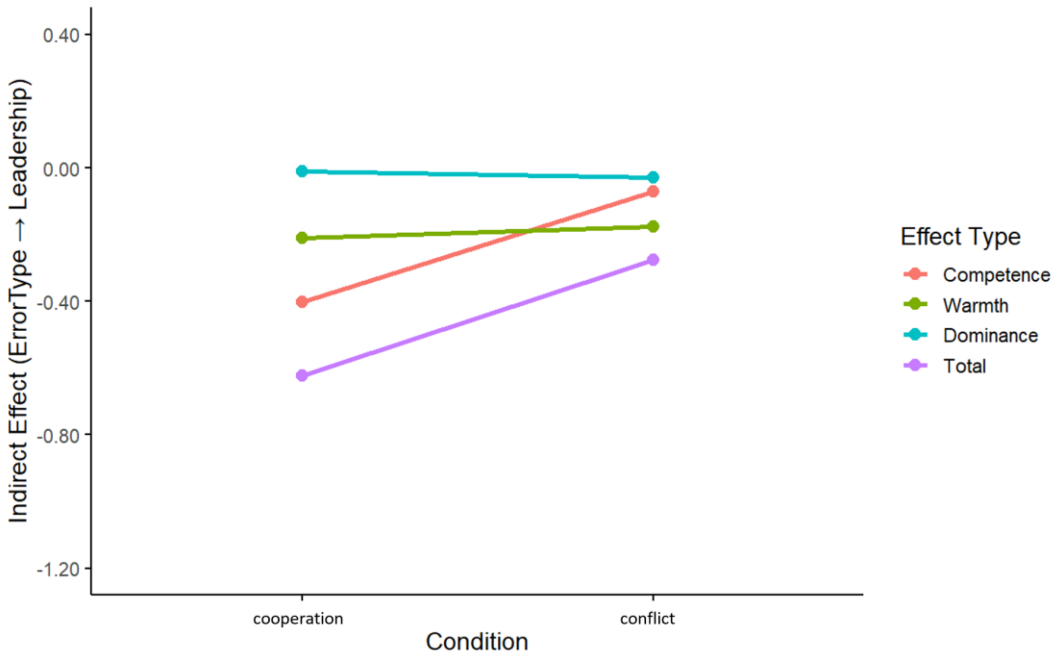

Study 4

Study 4 employed a 2 (error type: false positive vs. false negative) × 2 (cost: low vs. high) design in a war context. The model demonstrated an excellent fit to the data, $\chi^2(1) = 0.71$, $p = .400$, with *CFI* = 1.000, *TLI* = 1.020, *RMSEA* < .001 (90% *CI* [.000, .128]), and *SRMR* = .003. The model accounted for 1.3% of the variance in competence, 15.0% in warmth, 12.8% in dominance, and 37.8% in leadership preference.

Error type ("0" = false negative; "1" = false positive) significantly predicted warmth ($b = -0.52$, $p < .001$) and dominance ($b = 0.46$, $p = .005$), but not competence ($b = 0.05$, $p < .620$) or leadership preference ($b = -0.06$, $p = .678$). Cost ("0" = low; "1" = high) was not found predicting dominance ($b = -0.15$, $p = .335$), but marginal predicting warmth ($b = 0.19$, $p = .087$), competence ($b = 0.15$, $p = .069$), and

leadership preference (*b* = -0.20, *p* = .061). Leadership preference was significantly predicted by competence (*b* = 0.56, *p* < .001) and warmth (*b* = 0.64, *p* < .001), and dominance (*b* = 0.22, *p* < .001). A significant interaction term between error type and cost emerged for dominance (*b* = 0.45, *p* = .032), but not for warmth (*b* = -0.25, *p* = .132) and competence (*b* = -0.09, *p* = .438).

Although the index of moderated mediation was not significant, Δ*b* = -0.12, 95% *CI* [-0.45, 0.22], a pattern emerged: in the high-cost condition, the indirect effect of error type on leadership preference via reputation was significant (*b* = -0.32, 95% *CI* [-0.60, -0.06]), whereas in the low-cost condition, the indirect effect was not significant (*b* = -0.21, 95% *CI* [-0.51, 0.08]). This suggests that when the cost is high, false-positive leaders are less preferred, partly because of their reduced general reputation, whereas in the low-cost condition, such a mechanism was not found significant.

Different from Study 2 and 3, where perceived warmth and competence mattered, the difference in the total indirect effect was primarily driven by the combined indirect effect through perceived dominance and warmth. Specifically, the indirect effect through warmth was similarly significant in both the low-cost (*b* = -0.34, 95% *CI* [-0.56, -0.16]) and high-cost conditions (*b* = -0.50, 95% *CI* [-0.70, -0.33]), with no significant difference between the two (Δ*b* = -0.16, 95% *CI* [-0.38, 0.05]). For perceived dominance, the indirect effect in the high-cost condition (*b* = 0.20, 95% *CI* [-0.07, 0.36]) was significantly larger than that in the low-cost condition (*b* = 0.10, 95% *CI* [0.02, 0.21]), Δ*b*=0.10, 95% *CI* [0.06, 0.24]. The indirect effect via competence was not significant in either the low-cost (*b* = 0.03, 95% *CI* [-0.07, 0.14]) or high-cost condition (*b* = -0.03, 95% *CI* [-0.12, 0.06]), Δ*b* = -0.05, 95% *CI* [-0.21, 0.08]). In sum, during wars, the cost of a false negative did not associate with the preference for false-positive leaders via perceived warmth and competence, but rather with

perceived dominance. False-positive leaders were perceived as more dominant and were consequently more preferred as leaders. These indirect effects are plotted in Figure 4.

Figure 4

Indirect effects (error type to leadership preference)

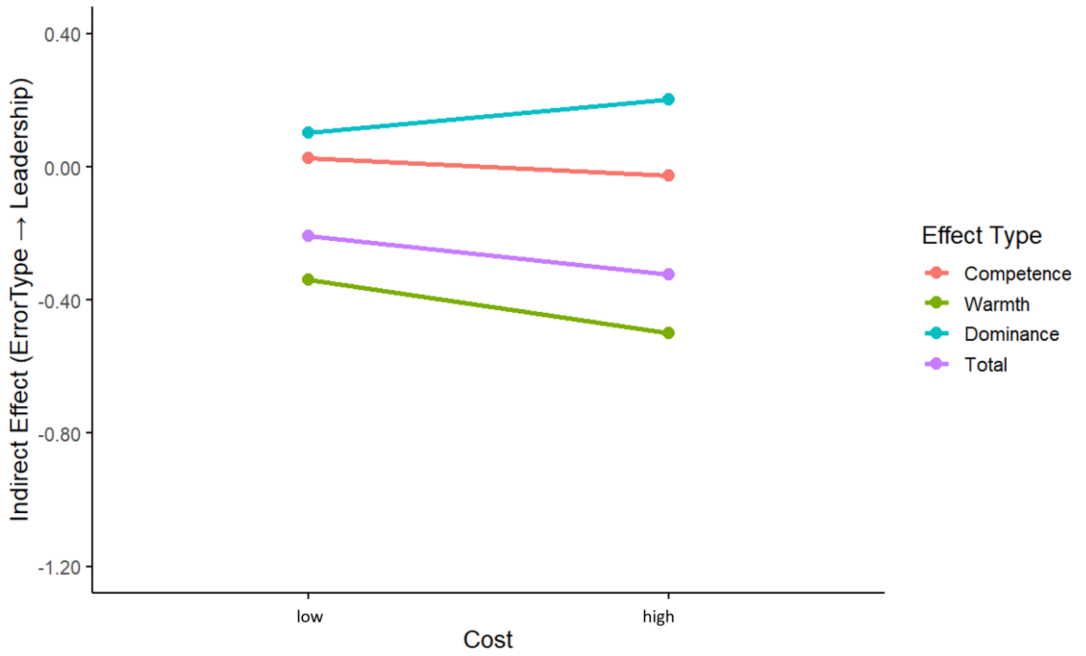

Discussion

The moderated mediation models across the three studies revealed several patterns:

1) Participants' preference for false-positive (vs. false-negative) leaders depended on leaders' general reputation, including perceived warmth, competence, and dominance;

2) When conflict was absent or cooperation was required, false-positive leaders were evaluated as having lower reputation, which associated with reduced leadership preference. However, when conflict was present, the preference for false-positive (vs. false-negative) leaders was not related to their reputation;

3) During conflict in which the cost of false-negative errors was high, false-positive leaders were less preferred due to their lower general reputation. By contrast, in the low-cost condition, leader preference for false-positive (vs. false-negative) leaders was not related reputation;

4) Among perceived warmth, competence, and dominance, warmth and competence emerged as mediators of leadership preference in conflict-absent and cooperation conditions, whereas warmth and dominance played a key role during conflict when the cost of false-negative errors was highlighted.

5) In all conditions, perceived warmth was a consistent mediator: false-positive leaders were reliably seen as less warm, which partly reduced people's leadership preference;

6) In conflict-absent and cooperation conditions, false-positive (vs. false-negative) leaders were perceived as less competent, which partly related to reduced leadership preference; however, in conflict conditions, false-positive (vs. false-negative) leaders were perceived as similarly competent, and leadership preference was therefore not related to perceived competence.

7) The mediating role of perceived dominance emerged when the cost of false-negative errors was emphasized, particularly in the high-cost (vs. low-cost) condition, where the preference for false-positive leaders partly related to increased perceived dominance.

These patterns reflected that leader preference was contingent on situational demands and mediated by general reputation (i.e., warmth, competence, and dominance). Preference against false-positive leaders was associated with reduced perceptions of general reputation, especially warmth and competence, which was in line with

previous studies (e.g., Laustsen & Bor, 2017). In contrast, general reputation mattered less during intergroup conflict, but cost considerations shifted which trait drove judgments. In high-cost conditions, lower perceived warmth still penalized preference for false-positive leaders, but the decrease was partially associated with higher perceived dominance. These findings suggested that false-positive leaders are not always disliked; instead, they may be either penalized or rewarded based on the existence of conflict and the magnitude of the false-negative cost.
