# Supplementary material for: Sharing conspiracy theories and staying in power: How leaders' false theories influence leadership perception
Source: Br J Soc Psychol. 2026 Apr 28;65:e70088. doi: 10.1111/bjso.70088 (PMC13125733; doi:10.1111/bjso.70088)
Supplement: Supplementary file 1 — Data S1. Supporting Information. [file BJSO-65-0-s001.zip › Study 1/Materials_S1.docx]

**Scenarios**

**Hit:**

Please imagine that you are a member of a tribe living in the Amazon Rainforest. Your tribe lives close to another tribe. The two tribes rely on the same resources for their living, but there is not always enough food for both tribes in this territory. So, your tribe is in constant competition with the other tribe. In the long run, only one of the two tribes can probably survive in this environment; the other will either have to leave the territory or run the risk of being disbanded.

Recently, some of your tribe members have died of bites by poisonous snakes. This is strange because there never used to be any poisonous snakes in this part of the Amazon rainforest.

During a tribal meeting, Aru, the leader of your tribe, stood up and had this to say:


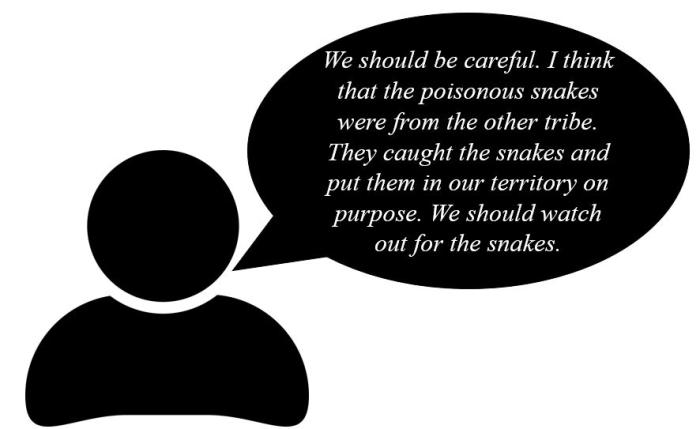


A few weeks later, your tribe members caught someone from the enemy tribe while he was releasing poisonous snakes into your territory, after which no snakes were found in your tribe anymore. It seems that your leader Aru did get the information right: Members of the other tribe have put the snakes in your territory on purpose.

**False positive**

Please imagine that you are a member of a tribe living in the Amazon Rainforest. Your tribe lives close to another tribe. The two tribes rely on the same resources for their living, but there is not always enough food for both tribes in this territory. So, your tribe is in constant competition with the other tribe. In the long run, only one of the two tribes can probably survive in this environment; the other will either have to leave the territory or run the risk of being disbanded.

 Recently, some of your tribe members have died of bites by poisonous snakes. This is strange because there never used to be any poisonous snakes in this part of the Amazon rainforest.

 During a tribal meeting, Aru, the leader of your tribe, stood up and had this to say:


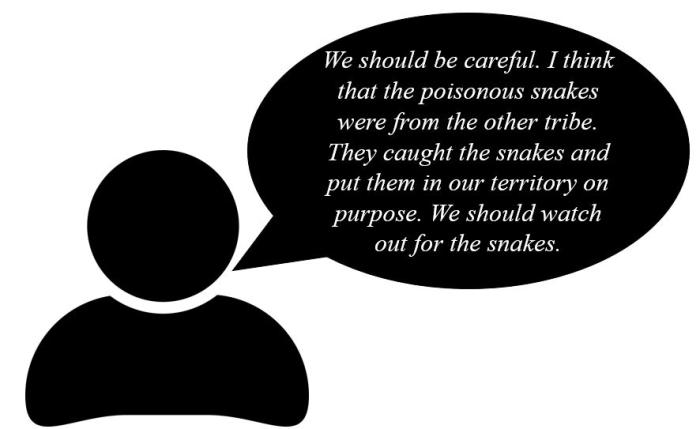


 A few weeks later, your tribe members caught the snakes while they were passing through your territory by coincidence, after which no snakes were found in your tribe anymore. It seems that your leader Aru did not get the information right: The snakes were not put by members of the other tribe on purpose but moved into your territory by accident.

**False negative**

Please imagine that you are a member of a tribe living in the Amazon Rainforest. Your tribe lives close to another tribe. The two tribes rely on the same resources for their living, but there is not always enough food for both tribes in this territory. So, your tribe is in constant competition with the other tribe. In the long run, only one of the two tribes can probably survive in this environment; the other will either have to leave the territory or run the risk of being disbanded.

 Recently, some of your tribe members have died of bites by poisonous snakes. This is strange because there never used to be any poisonous snakes in this part of the Amazon rainforest.

 During a tribal meeting, Aru, the leader of your tribe, stood up and had this to say:


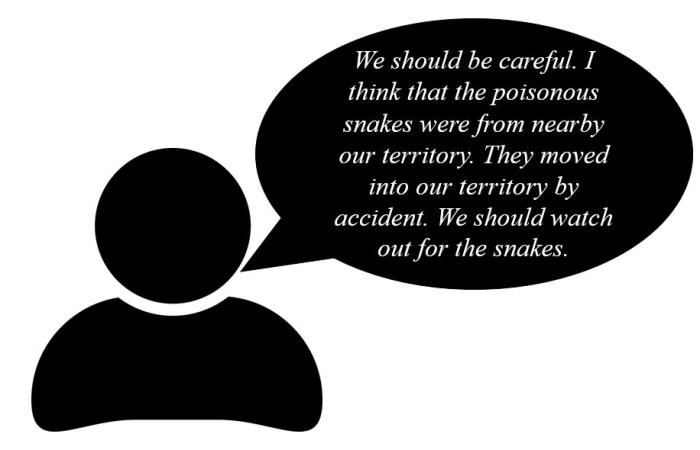


 A few weeks later, your tribe members caught someone from the enemy tribe while he was releasing poisonous snakes into your territory, after which no snakes were found in your tribe anymore. It seems that your leader Aru did not get the information right: The snakes did not move into your territory by accident but were put by members of the other tribe on purpose.

**Correct rejection**

Please imagine that you are a member of a tribe living in the Amazon Rainforest. Your tribe lives close to another tribe. The two tribes rely on the same resources for their living, but there is not always enough food for both tribes in this territory. So, your tribe is in constant competition with the other tribe. In the long run, only one of the two tribes can probably survive in this environment; the other will either have to leave the territory or run the risk of being disbanded.

 Recently, some of your tribe members have died of bites by poisonous snakes. This is strange because there never used to be any poisonous snakes in this part of the Amazon rainforest.

 During a tribal meeting, Aru, the leader of your tribe, stood up and had this to say:


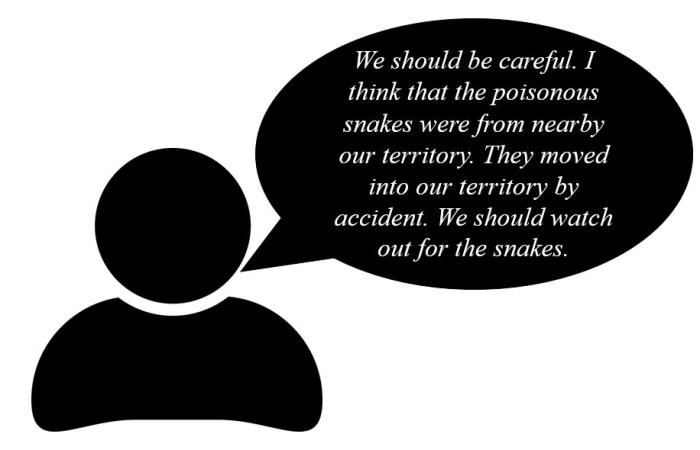


 A few weeks later, your tribe members caught the snakes while they were passing through your territory by coincidence, after which no snakes were found in your tribe anymore. It seems that your leader Aru did get the information right: The snakes were moved into your territory by accident

**Measurements**

How do you think of Aru?

| Dominance | 1-  Not at all | 2 | 3 | 4-Moderate | 5 | 6 | 7-  Very much |
| --- | --- | --- | --- | --- | --- | --- | --- |
| I think Aru is dominant. |  |  |  |  |  |  |  |
| I think Aru is forceful. |  |  |  |  |  |  |  |
| I think Aru is influential. |  |  |  |  |  |  |  |
| I think Aru is powerful. |  |  |  |  |  |  |  |
| I think Aru is self-assured. |  |  |  |  |  |  |  |

How do you think of Aru?

| Competence | 1  -Not at all | 2 | 3 | 4  -Neutral | 5 | 6 | 7  -Very much |
| --- | --- | --- | --- | --- | --- | --- | --- |
| Aru has much potential. |  |  |  |  |  |  |  |
| Aru performs very well at a number of things. |  |  |  |  |  |  |  |
| Aru is talented. |  |  |  |  |  |  |  |
| Aru is not very competent. |  |  |  |  |  |  |  |
| Aru deals poorly with challenges. |  |  |  |  |  |  |  |
| Please select "Not at all" |  |  |  |  |  |  |  |

I think that Aru

| Warmth | 1  -Not at all | 2 | 3 | 4  -Neutral | 5 | 6 | 7  -Very much |
| --- | --- | --- | --- | --- | --- | --- | --- |
| Is helpful and unselfish with others. |  |  |  |  |  |  |  |
| Has a forgiving nature. |  |  |  |  |  |  |  |
| Can be cold and aloof. |  |  |  |  |  |  |  |
| Is considerate and kind to almost everyone. |  |  |  |  |  |  |  |
| Likes to cooperate with others. |  |  |  |  |  |  |  |
