# Supplementary material for: Sharing conspiracy theories and staying in power: How leaders' false theories influence leadership perception
Source: Br J Soc Psychol. 2026 Apr 28;65:e70088. doi: 10.1111/bjso.70088 (PMC13125733; doi:10.1111/bjso.70088)
Supplement: Supplementary file 1 — Data S1. Supporting Information. [file BJSO-65-0-s001.zip › Study 2/Material_study2.docx]

False positive + Conflict-present

Please imagine that you are a member of a tribe living in the Amazon Rainforest. Your tribe lives close to another tribe. The two tribes rely on the same resources for their living, but there is not always enough food for both tribes in this territory. So, your tribe is in constant competition with the other tribe. In the long run, only one of the two tribes can probably survive in this environment; the other will either have to leave the territory or run the risk of being disbanded.

Recently, some of your tribe members have died of bites by poisonous snakes. This is strange because there never used to be any poisonous snakes in this part of the Amazon rainforest.

During a tribal meeting, Aru, the leader of your tribe, stood up and had this to say:


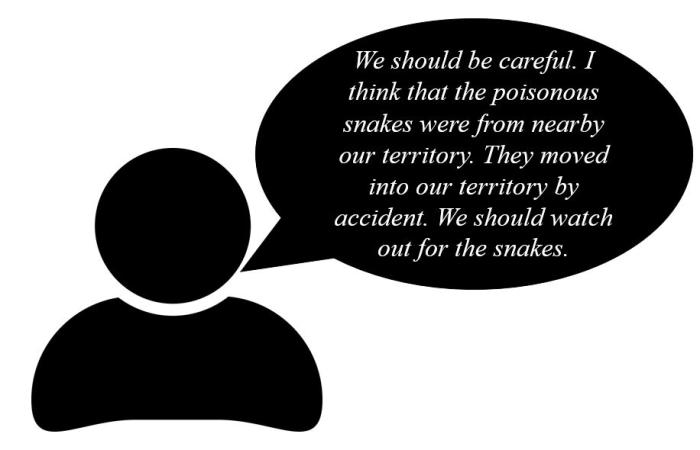


 A few weeks later, your tribe members caught someone from the other tribe while he was releasing poisonous snakes into your territory, after which no snakes were found in your tribe anymore. **It seems that your leader Aru did not get the information right: The snakes did not move into your territory by accident but were put by members of the other tribe on purpose.**

False positive + Conflict-absent

Please imagine that you are a member of a tribe living in the Amazon Rainforest. Your tribe lives close to another tribe. The two tribes rely on the same resources for their living, and there is enough food for both tribes in this territory. They have no communication or conflicts with each other. In the long run, both tribes can probably survive in this environment without having any contact with each other; neither tribe will have to leave the territory or runs the risk of being disbanded.

 Recently, some of your tribe members have died of bites by poisonous snakes. This is strange because there never used to be any poisonous snakes in this part of the Amazon rainforest.

 During a tribal meeting, Aru, the leader of your tribe, stood up and had this to say:


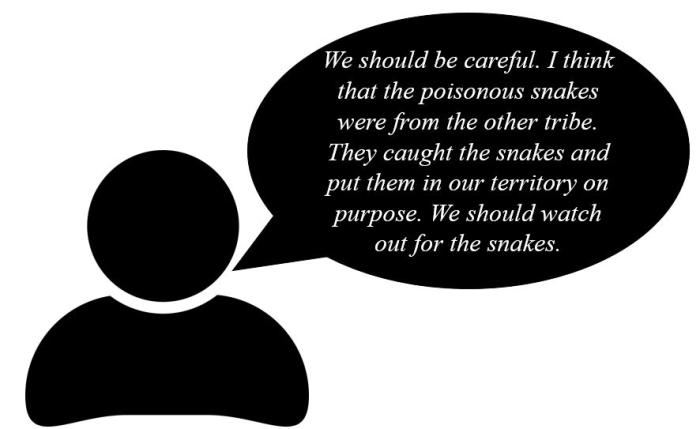


A few weeks later, your tribe members caught the snakes while they were passing through your territory by coincidence, after which no snakes were found in your tribe anymore. **It seems that your leader Aru did not get the information right: The snakes were not put by members of the other tribe on purpose but moved into your territory by accident.**

False negative + Conflict-present

Please imagine that you are a member of a tribe living in the Amazon Rainforest. Your tribe lives close to another tribe. The two tribes rely on the same resources for their living, but there is not always enough food for both tribes in this territory. So, your tribe is in constant competition with the other tribe. In the long run, only one of the two tribes can probably survive in this environment; the other will either have to leave the territory or run the risk of being disbanded.

Recently, some of your tribe members have died of bites by poisonous snakes. This is strange because there never used to be any poisonous snakes in this part of the Amazon rainforest.

During a tribal meeting, Aru, the leader of your tribe, stood up and had this to say:


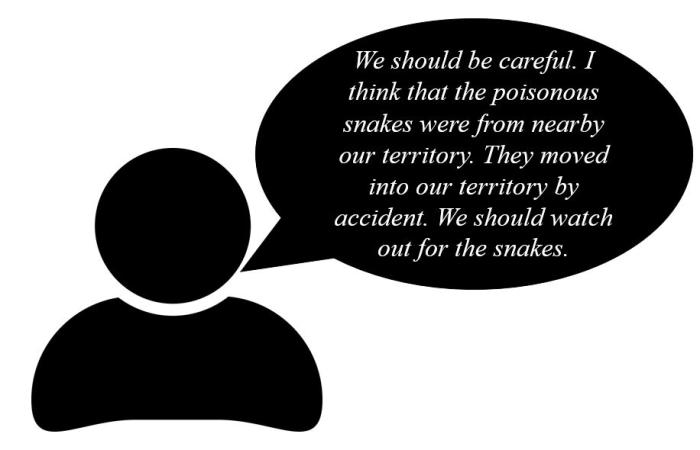


 A few weeks later, your tribe members caught someone from the other tribe while he was releasing poisonous snakes into your territory, after which no snakes were found in your tribe anymore. **It seems that your leader Aru did not get the information right: The snakes did not move into your territory by accident but were put by members of the other tribe on purpose.**

False negative + Conflict-absent

Please imagine that you are a member of a tribe living in the Amazon Rainforest. Your tribe lives close to another tribe. The two tribes rely on the same resources for their living, and there is enough food for both tribes in this territory. They have no communication or conflicts with each other. In the long run, both tribes can probably survive in this environment without having any contact with each other; neither tribe will have to leave the territory or runs the risk of being disbanded.

Recently, some of your tribe members have died of bites by poisonous snakes. This is strange because there never used to be any poisonous snakes in this part of the Amazon rainforest.
 
During a tribal meeting, Aru, the leader of your tribe, stood up and had this to say:


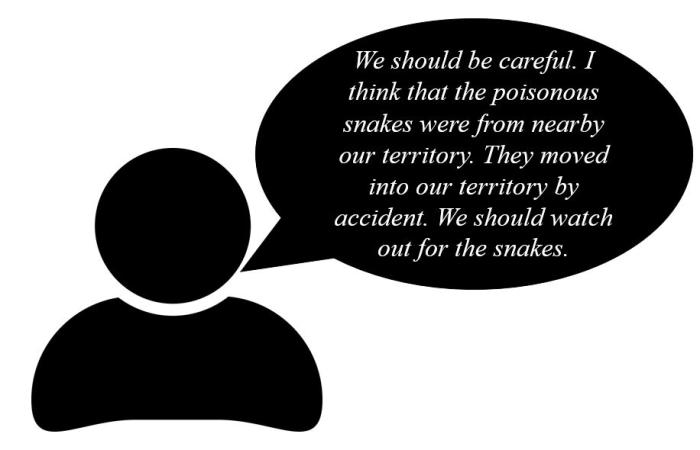


 A few weeks later, your tribe members caught someone from the other tribe while he was releasing poisonous snakes into your territory, after which no snakes were found in your tribe anymore. **It seems that your leader Aru did not get the information right: The snakes did not move into your territory by accident but were put by members of the other tribe on purpose.**
