# Supplementary material for: Sharing conspiracy theories and staying in power: How leaders' false theories influence leadership perception
Source: Br J Soc Psychol. 2026 Apr 28;65:e70088. doi: 10.1111/bjso.70088 (PMC13125733; doi:10.1111/bjso.70088)
Supplement: Supplementary file 1 — Data S1. Supporting Information. [file BJSO-65-0-s001.zip › Study 3/Material_Study3.docx]

False positive + Cooperation

Please imagine that you are a devoted member of an e-commerce company. Your company is in a fruitful cooperation with another company named Loco, jointly vying for market supremacy. Now, at the most critical juncture of this cooperation, both companies have implemented numerous strategies to help each other's expansion. In the long run, it's clear that by helping each other, both companies can stay on top of the market.

 Three days ago, your company hosted a crucial commercial event, which has consistently accounted for a significant portion of its annual profits. However, an unforeseen incident transpired, causing immense distress and uncertainty. The company's IT system, which had always functioned seamlessly in the past, unexpectedly experienced a failure just hours before the event was scheduled to kick off. Customers remained unable to access the online shop's website for three hours beyond the promised start time. This resulted in significant profit losses and customer complaints. None of your colleagues possess any insight into the root cause of this system breakdown.
  
During the emergency meeting of your company, John, the head of your department, stood up and had this to say:


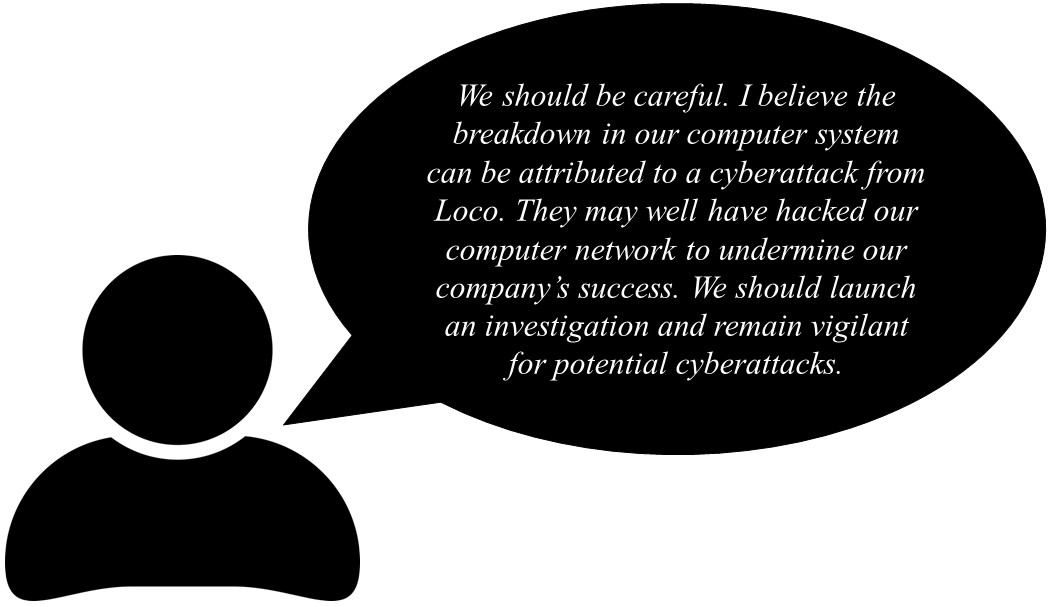


After a few days of investigation, some bugs in the system were found, which were the reasons for the system breakdown. It seems that your leader John did not get the information right: The breakdown was **not** because of the cyberattack from Loco **but** because of bugs in the system.

False negative + Cooperation

Please imagine that you are a devoted member of an e-commerce company. Your company is in a fruitful cooperation with another company named Loco, jointly vying for market supremacy. Now, at the most critical juncture of this cooperation, both companies have implemented numerous strategies to help each other's expansion. In the long run, it's clear that by helping each other, both companies can stay on top of the market.

Three days ago, your company hosted a crucial commercial event, which has consistently accounted for a significant portion of its annual profits. However, an unforeseen incident transpired, causing immense distress and uncertainty. The company's IT system, which had always functioned seamlessly in the past, unexpectedly experienced a failure just hours before the event was scheduled to kick off. Customers remained unable to access the online shop's website for three hours beyond the promised start time. This resulted in significant profit losses and customer complaints. None of your colleagues possess any insight into the root cause of this system breakdown.
  
During the emergency meeting of your company, John, the head of your department, stood up and had this to say:

  
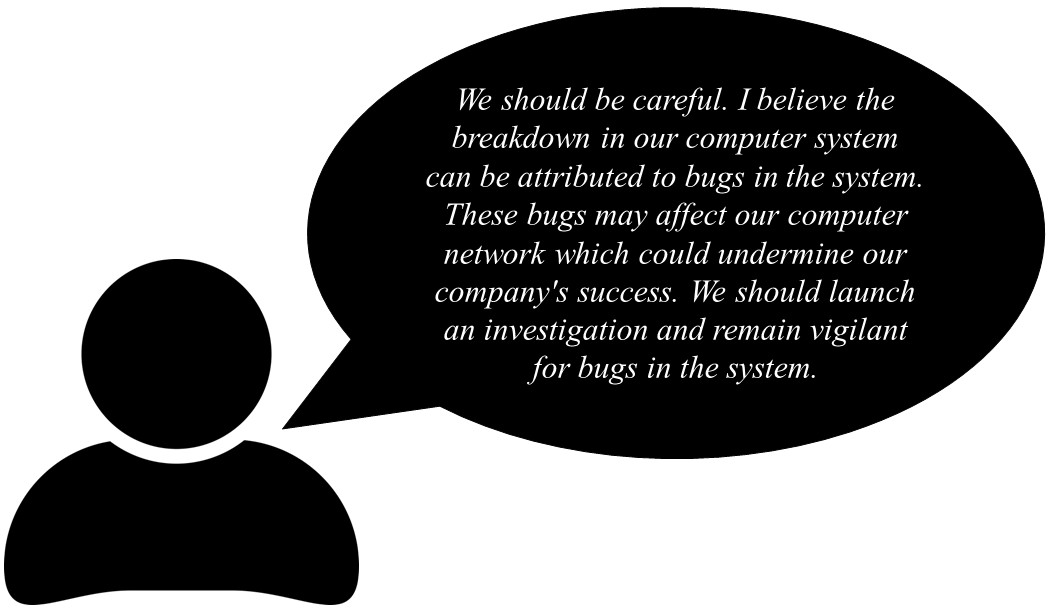


After a few days of investigation, some evidence of a cyberattack from Loco was found, which was the reason for the system breakdown. It seems that your leader John did not get the information right: The breakdown was **not** because of bugs in the system **but** because of a cyberattack from Loco.

False positive + Conflict

Please imagine that you are a devoted member of an e-commerce company. Your company is in cutthroat competition with another company named Loco, vying for market supremacy. Now, at the most critical juncture of this competition, both companies have implemented numerous strategies to hinder each other's expansion. In the long run, only one of the two companies can stay on top of the market. The other is likely to face deteriorating financial conditions or even the threat of bankruptcy and restructuring.

 Three days ago, your company hosted a crucial commercial event, which has consistently accounted for a significant portion of its annual profits. However, an unforeseen incident transpired, causing immense distress and uncertainty. The company's IT system, which had always functioned seamlessly in the past, unexpectedly experienced a failure just hours before the event was scheduled to kick off. Customers remained unable to access the online shop's website for three hours beyond the promised start time. This resulted in significant profit losses and customer complaints. None of your colleagues have any insight into the root cause of this system breakdown. During the emergency meeting of your company, John, the head of your department, stood up and had this to say:
  
 During the emergency meeting of your company, John, the head of your department, stood up and had this to say:

   
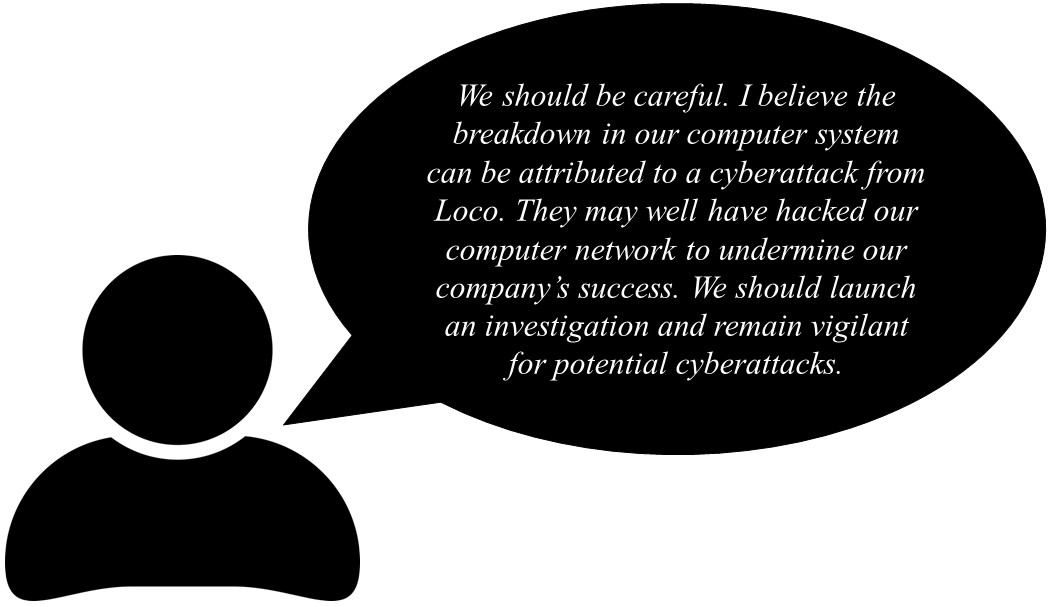


 After a few days of investigation, some bugs in the system were found, which were the reasons for the system breakdown. It seems that your leader John did not get the information right: The breakdown was **not** because of the cyberattack from Loco **but** because of bugs in the system.

False negative- Conflict

Please imagine that you are a devoted member of an e-commerce company. Your company is in cutthroat competition with another company named Loco, vying for market supremacy. Now, at the most critical juncture of this competition, both companies have implemented numerous strategies to hinder each other's expansion. In the long run, only one of the two companies can stay on top of the market. The other is likely to face deteriorating financial conditions or even the threat of bankruptcy and restructuring.

 Three days ago, your company hosted a crucial commercial event, which has consistently accounted for a significant portion of its annual profits. However, an unforeseen incident transpired, causing immense distress and uncertainty. The company's IT system, which had always functioned seamlessly in the past, unexpectedly experienced a failure just hours before the event was scheduled to kick off. Customers remained unable to access the online shop's website for three hours beyond the promised start time. This resulted in significant profit losses and customer complaints. None of your colleagues have any insight into the root cause of this system breakdown. During the emergency meeting of your company, John, the head of your department, stood up and had this to say:
  
 During the emergency meeting of your company, John, the head of your department, stood up and had this to say:


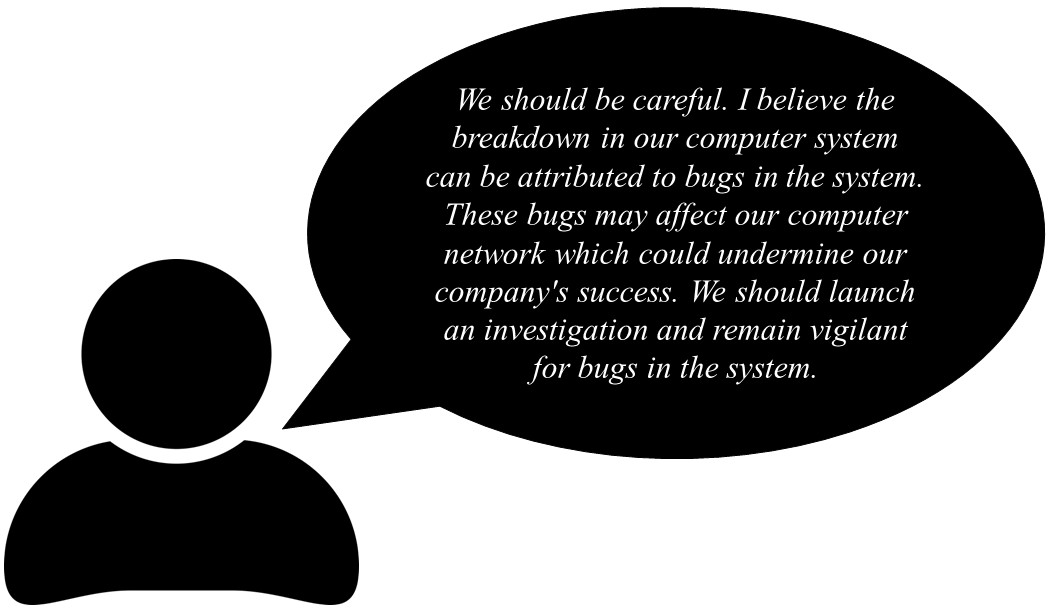

   

 After a few days of investigation, some evidence of a cyberattack from Loco was found, which was the reason for the system breakdown. It seems that your leader John did not get the information right: The breakdown was **not** because of bugs in the system **but** because of a cyberattack from Loco.
