# Supplementary material for: Sharing conspiracy theories and staying in power: How leaders' false theories influence leadership perception
Source: Br J Soc Psychol. 2026 Apr 28;65:e70088. doi: 10.1111/bjso.70088 (PMC13125733; doi:10.1111/bjso.70088)
Supplement: Supplementary file 1 — Data S1. Supporting Information. [file BJSO-65-0-s001.zip › Study 4/Material_Study4.docx]

False positive + High cost

Please imagine that you are a devoted member of one of the two parties in a country of Africa. Your party is in a conflict with another party, vying for control of the country. Now, at the most critical juncture of this conflict, the two parties have established their dominance over different regions within the country and have engaged in prolonged acts of violence against each other. In the long run, the winner will gain control of the entire country, while the losing party will face the option of surrender or the potential risk of deportation.

 A perplexing incident has recently occurred, causing great concern in your party. A military storage facility caught fire, resulting in a massive explosion and numerous fatalities. The circumstances surrounding this incident remain unclear, and none of your fellow party members have any knowledge as to what caused the fire. However, your party is experiencing a shortage of ammunition. If similar incidents occur in other military facilities, your party will risk losing crucial weapons necessary for future combat, potentially failing the conflict. The consequence is significant if your party misidentifies the cause of the fire.

 During a party conference, John, the leader of your party, stood up and had this to say:


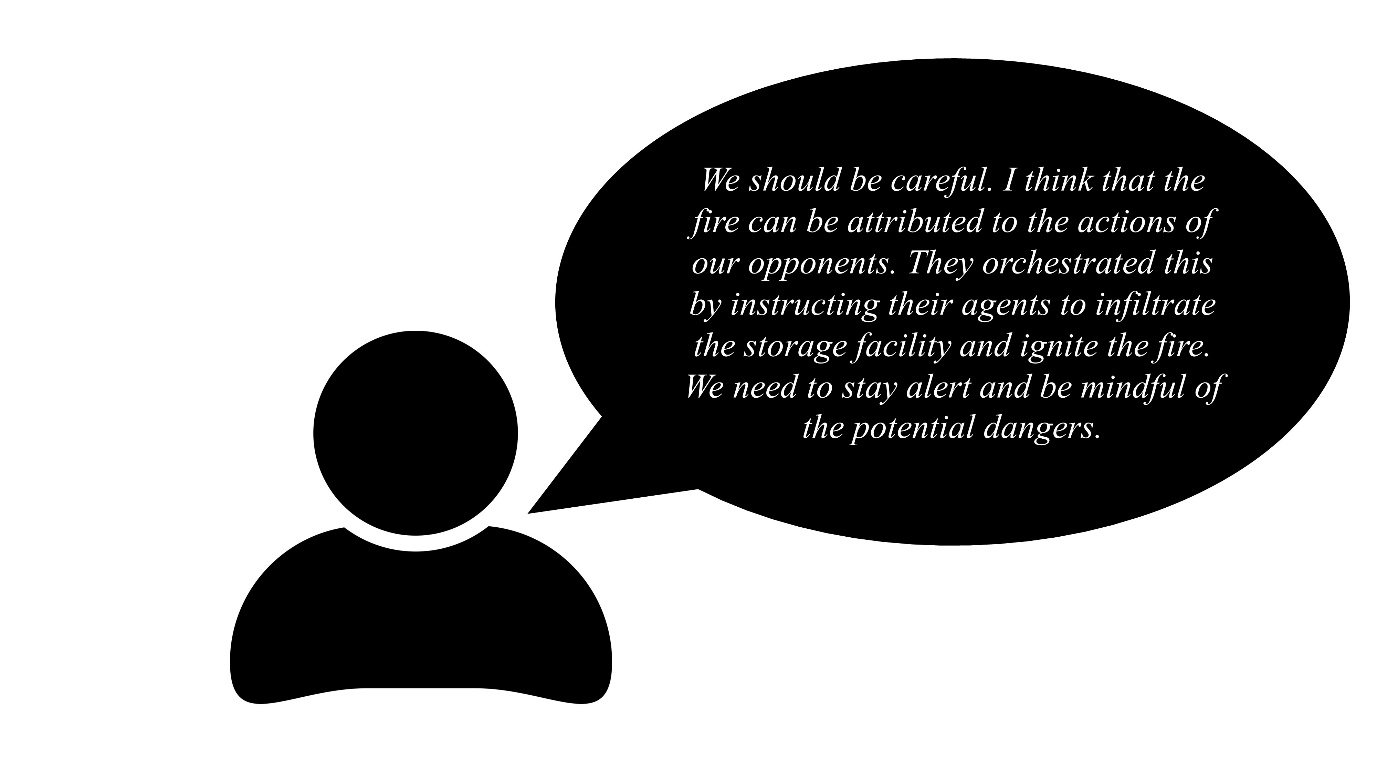


 After several days of investigation, no evidence of human involvement in the fire was discovered. Meanwhile, experts uncovered clear indications that the fire resulted from escalating temperatures and dry conditions. It seems that your leader John did not get the information right: The fire in the storage was not set by the agents of the other party but because of the high temperature and dry conditions.

False positive + Low cost

Please imagine that you are a devoted member of one of the two parties in a country of Africa. Your party is in a conflict against another party, vying for control of the country. Now, at the most critical juncture of this conflict, the two parties have established their dominance over different regions within the country and have engaged in prolonged acts of violence against each other. In the long run, it’s that the winner will gain control of the entire country, while the losing party will face the option of surrender or the potential risk of deportation.

 A perplexing incident has recently occurred, causing great concern in your party. A military storage facility caught fire, resulting in a massive explosion and numerous fatalities. The circumstances surrounding this incident remain unclear, and none of your fellow party members have any knowledge as to what caused the fire. However, your party has a good supply of ammunition. Even if similar incidents occur in other military facilities, your party will not risk losing crucial weapons necessary for future combat. The consequence is small if your party misidentifies the cause of the fire.

During a party conference, John, the leader of your party, stood up and had this to say:


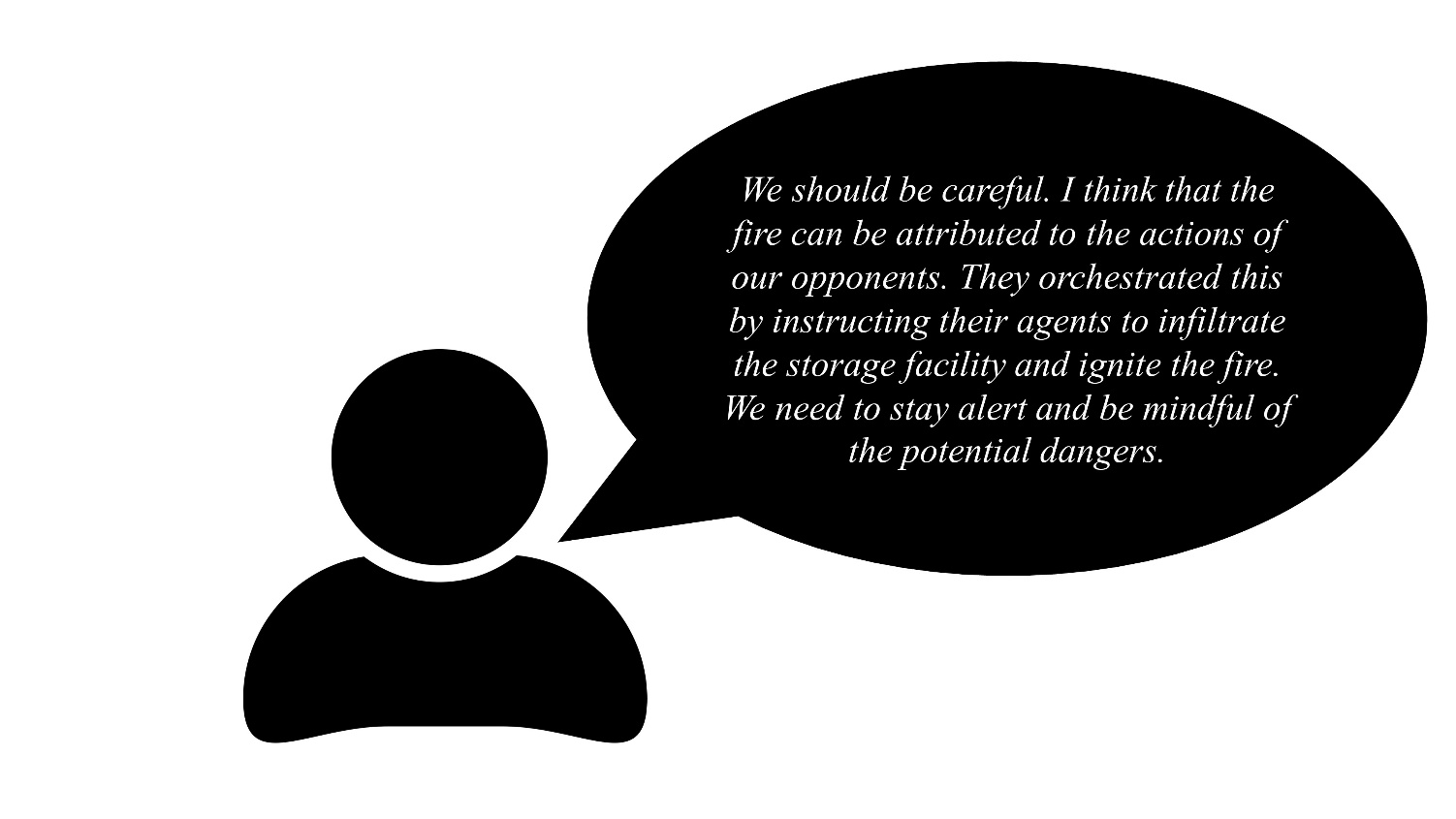


After several days of investigation, no evidence of human involvement in the fire was discovered. Meanwhile, experts uncovered clear indications that the fire resulted from escalating temperatures and dry conditions. It seems that your leader John did not get the information right: The fire in the storage was not set by the agents of the other party but because of the high temperature and dry conditions.

False negative + High cost

Please imagine that you are a devoted member of one of the two parties in a country of Africa. Your party is in a conflict against another party, vying for control of the country. Now, at the most critical juncture of this conflict, the two parties have established their dominance over different regions within the country and have engaged in prolonged acts of violence against each other. In the long run, it’s that the winner will gain control of the entire country, while the losing party will face the option of surrender or the potential risk of deportation.

 A perplexing incident has recently occurred, causing great concern in your party. A military storage facility caught fire, resulting in a massive explosion and numerous fatalities. The circumstances surrounding this incident remain unclear, and none of your fellow party members have any knowledge as to what caused the fire. However, your party is experiencing a shortage of ammunition. If similar incidents occur in other military facilities, your party will risk losing crucial weapons necessary for future combat, potentially failing the conflict. The consequence is significant if your party misidentifies the cause of the fire.

 During a party conference, John, the leader of your party, stood up and had this to say:


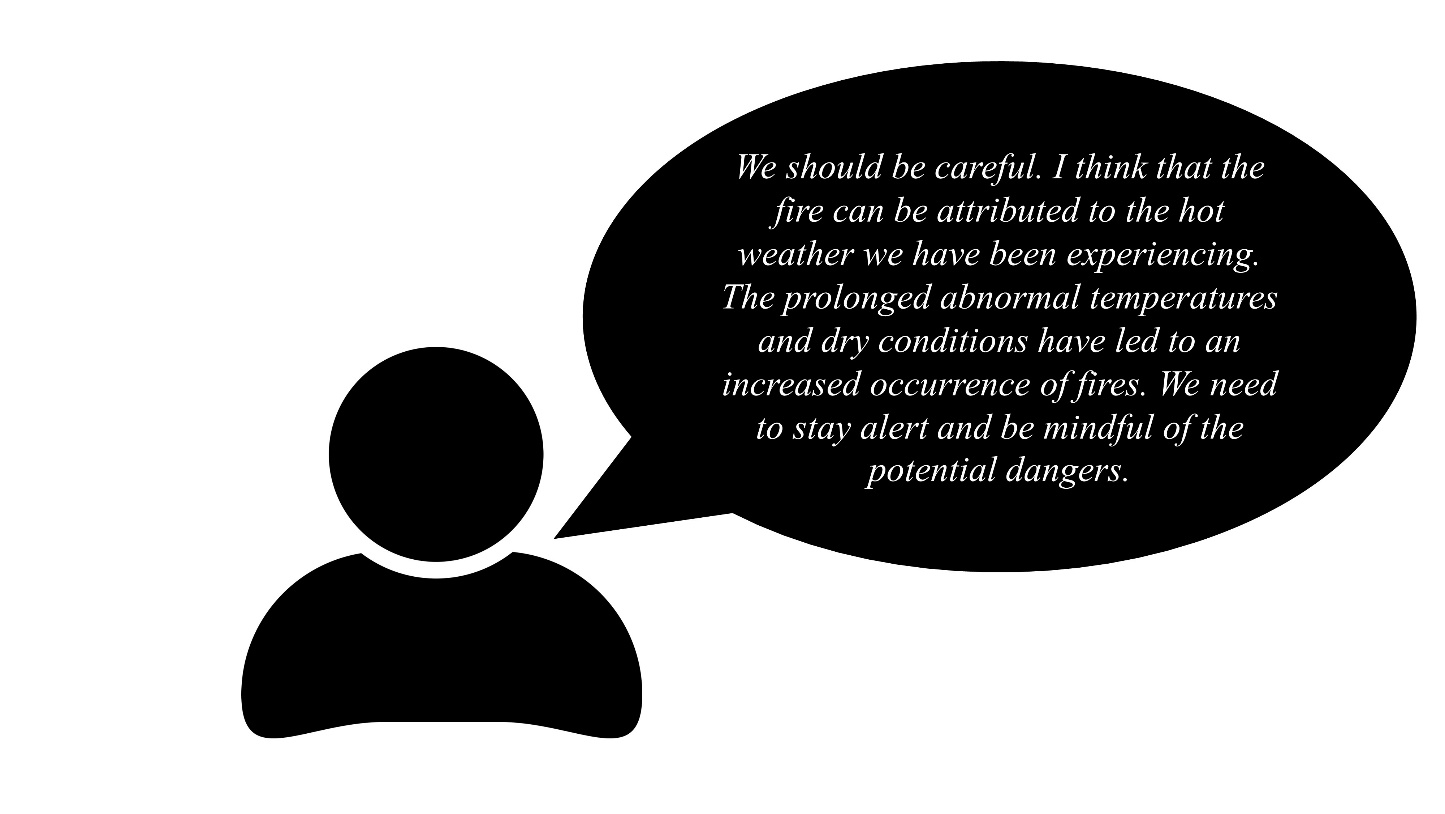

  
 After several days of investigation, evidence of human involvement in the fire was found. Meanwhile, soldiers of your party captured the agents of the other party while they were trying to set fire to another military facility. It seems that your leader John did not get the information right: The fire was not because of the high temperature and dry conditions but because of the agents of the other party.

False negative + Low cost

Please imagine that you are a devoted member of one of the two parties in a country of Africa. Your party is in a conflict against another party, vying for control of the country. Now, at the most critical juncture of this conflict, the two parties have established their dominance over different regions within the country and have engaged in prolonged acts of violence against each other. In the long run, it’s that the winner will gain control of the entire country, while the losing party will face the option of surrender or the potential risk of deportation.

 A perplexing incident has recently occurred, causing great concern in your party. A military storage facility caught fire, resulting in a massive explosion and numerous fatalities. The circumstances surrounding this incident remain unclear, and none of your fellow party members have any knowledge as to what caused the fire. However, your party has a good supply of ammunition. Even if similar incidents occur in other military facilities, your party will not risk losing crucial weapons necessary for future combat. The consequence is small if your party misidentifies the cause of the fire.

 During a party conference, John, the leader of your party, stood up and had this to say:


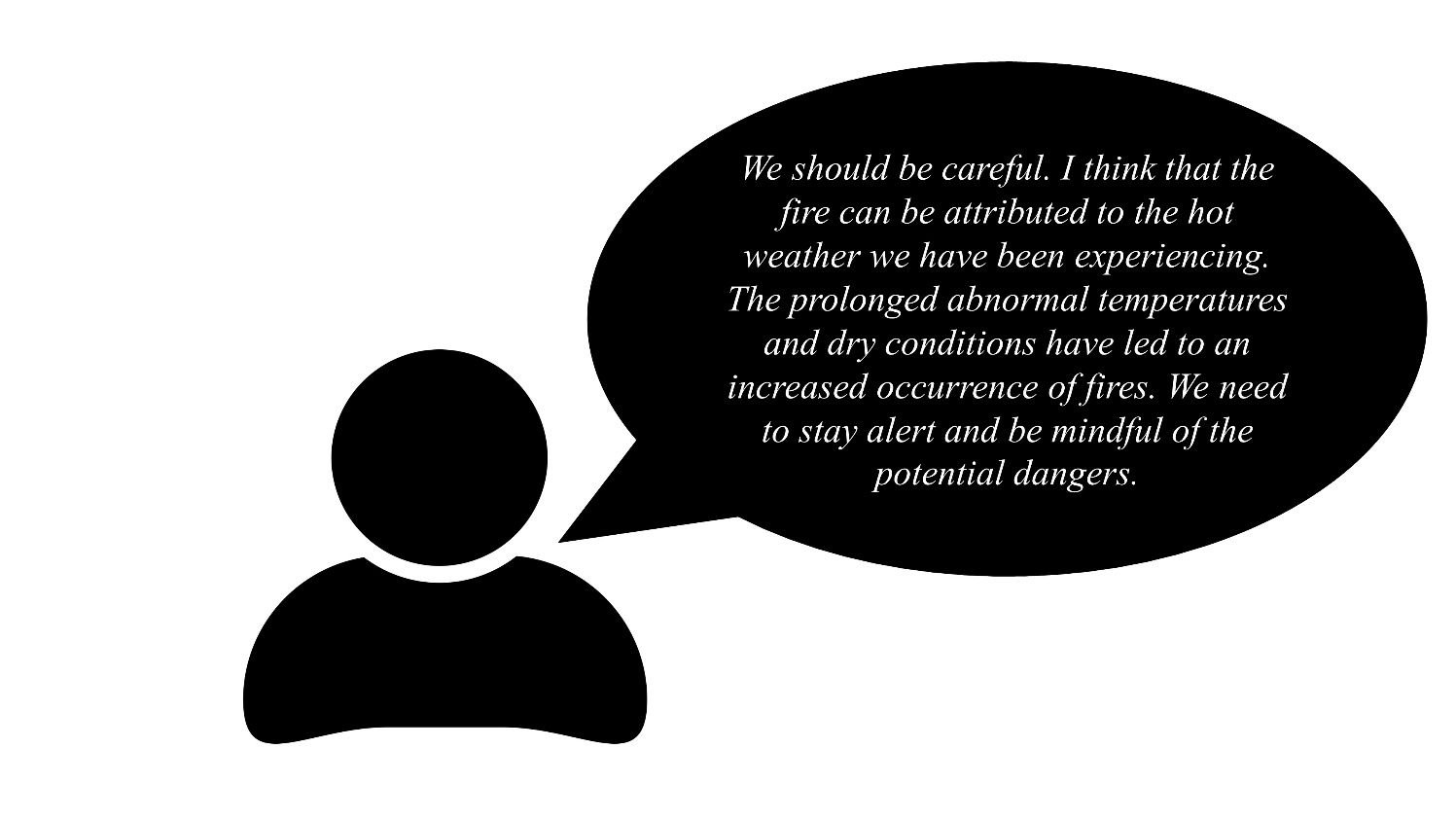


After several days of investigation, evidence of human involvement in the fire was found. Meanwhile, soldiers of your party captured the agents of the other party while they were trying to set fire to another military facility. It seems that your leader John did not get the information right: The fire was not because of the high temperature and dry conditions but because of the agents of the other party.
